# Benzimidazole Derivatives as New and Selective Inhibitors of Arginase from *Leishmania mexicana* with Biological Activity against Promastigotes and Amastigotes

**DOI:** 10.3390/ijms222413613

**Published:** 2021-12-19

**Authors:** Irene Betancourt-Conde, Claudia Avitia-Domínguez, Alicia Hernández-Campos, Rafael Castillo, Lilián Yépez-Mulia, Jesús Oria-Hernández, Sara T. Méndez, Erick Sierra-Campos, Mónica Valdez-Solana, Siseth Martínez-Caballero, Juan A. Hermoso, Antonio Romo-Mancillas, Alfredo Téllez-Valencia

**Affiliations:** 1 Facultad de Medicina y Nutrición, Universidad Juárez del Estado de Durango, Av. Universidad y Fanny Anitúa S/N, Durango 34000, Mexico; betancourtirene@gmail.com; 2 Departamento de Farmacia, Facultad de Química, Universidad Nacional Autónoma de México, Mexico City 04510, Mexico; hercam@unam.mx (A.H.-C.); rafaelc@unam.mx (R.C.); 3 Unidad de Investigación Médica en Enfermedades Infecciosas y Parasitarias, Unidad Médica de Alta Especialidad-Hospital de Pediatría, Centro Médico Nacional Siglo XXI, Instituto Mexicano del Seguro Social, Mexico City 06720, Mexico; lilianyepez@yahoo.com; 4 Laboratorio de Bioquímica-Genética, Instituto Nacional de Pediatría, Secretaría de Salud, Mexico City 04530, Mexico; jesus.oria.inp@gmail.com (J.O.-H.); saratme@hotmail.com (S.T.M.); 5 Facultad de Ciencias Químicas, Universidad Juárez del Estado de Durango, Av. Artículo 123 S/N Fracc. Filadelfia, Gómez Palacio 35010, Mexico; ericksier@ujed.mx (E.S.-C.); valdezandyval@gmail.com (M.V.-S.); 6 Departamento de Cristalografía y Biología Estructural, Instituto Química-Física Rocasolano, Consejo Superior de Investigaciones Científicas, 28006 Madrid, Spain; siseth.martinez@iquimica.unam.mx (S.M.-C.); xjuan@iqfr.csic.es (J.A.H.); 7 Laboratorio de Diseño Asistido por Computadora y Síntesis de Fármacos, Facultad de Química, Universidad Autónoma de Querétaro, Querétaro 76010, Mexico; ruben.romo@uaq.mx

**Keywords:** leishmaniasis, arginase, virtual screening, enzyme inhibition, molecular dynamics, benzimidazole derivatives

## Abstract

Leishmaniasis is a disease caused by parasites of the *Leishmania* genus that affects 98 countries worldwide, 2 million of new cases occur each year and more than 350 million people are at risk. The use of the actual treatments is limited due to toxicity concerns and the apparition of resistance strains. Therefore, there is an urgent necessity to find new drugs for the treatment of this disease. In this context, enzymes from the polyamine biosynthesis pathway, such as arginase, have been considered a good target. In the present work, a chemical library of benzimidazole derivatives was studied performing computational, enzyme kinetics, biological activity, and cytotoxic effect characterization, as well as in silico ADME-Tox predictions, to find new inhibitors for arginase from *Leishmania mexicana* (LmARG). The results show that the two most potent inhibitors (compounds **1** and **2**) have an I_50_ values of 52 μM and 82 μM, respectively. Moreover, assays with human arginase 1 (HsARG) show that both compounds are selective for LmARG. According to molecular dynamics simulation studies these inhibitors interact with important residues for enzyme catalysis. Biological activity assays demonstrate that both compounds have activity against promastigote and amastigote, and low cytotoxic effect in murine macrophages. Finally, in silico prediction of their ADME-Tox properties suggest that these inhibitors support the characteristics to be considered drug candidates. Altogether, the results reported in our study suggest that the benzimidazole derivatives are an excellent starting point for design new drugs against leishmanisis.

## 1. Introduction

Leishmaniasis is a neglected tropical disease transmitted by sandfly vector (*Phlebotomus* or *Lutzomyia*) via anthroponotic or zoonotic cycles [1]. The three main forms of leishmaniasis include cutaneous (CL), mucosal (MCL) and visceral leishmaniasis (VL) [2]. This disease affects 98 countries, and it is estimated that approximately 2 million of new cases occur each year and more than 350 million people are at risk [3].

The first line treatment against the leishmaniasis consist in sodium stibogluconate (Pentostam) and meglumine antimoniate (Glucantime) [4]. Nevertheless, their use is limited due to their toxicity and the apparition of resistance strains [5]. Pentamidine and Amphotericin B are used as second line drugs, but as well as antimonials, their use is limited due to its toxicity and the parasite resistance [6,7]. Miltefosine is the only existing oral drug to the treatment of leishmaniasis, however, its use has not been satisfactory due to the potential parasite resistance, also it is necessary to continue with its pharmacovigilance [8]. Other proposed topical treatments involve Rifamycin and novel drug-delivery systems that may help the diffusion and the efficacy [9]. Recently, a combined therapy strategy has been used to overcome the resistance in bacteria, nevertheless, this has been poorly tested in leishmaniasis and it must be established for each species of *Leishmania* [10]. Therefore, it is important to look for new drugs against leishmaniasis.

In this regard, because of their central role in proliferation, differentiation, and antioxidant mechanisms in *Leishmania*, enzymes for polyamines (PAs) biosynthesis are considered attractive drug targets to develop new drugs against leishmaniasis. Moreover, this pathway is linked with the trypanothione pathway and its enzymes are structurally different from their homologous in human [11,12]. The PAs biosynthesis pathway starts with the synthesis of the precursor L-ornithine, catalyzed by arginase (ARG) through the hydrolysis of L-arginine. Afterwards, ornithine is transformed to putrescine by ornithine decarboxylase. Finally, spermidine synthase converts putrescine in spermidine, which is one of the precursors for trypanothione biosynthesis [12,13,14]. One of the enzymes that has been received a special interest is arginase.

Structurally, arginase from *Leishmania mexicana* (LmARG) is a homotrimer stabilized by intermolecular ionic interactions and hydrogen bonds. This enzyme belongs to α/β family and is composed by eight-stranded β-sheet flanked on both sides by α-helices. Arginase is a metalloenzyme that requires of two manganese ions for its enzyme activity arrayed as a binuclear Mn^+2^ center located at the bottom of a ~15 Å-deep active site cleft coordinated in an octahedral arrangement. The catalytic residues are Glu288 which participates in substrate recognition, whilst Asp141 stabilizes the hydroxide ion, and His139 that is a proton shuttle [15].

Despite of their similar folding with the human arginase 1 (HsARG), some differences are shown in the tertiary structure. Three regions change in the LmARG, first, a new helix was found (Gly62-Ser71), which corresponds to a loop in the HsARG. Also, the loop formed by residues Leu161-Cys172, and helix formed by Ile219-Val230 in HsARG (LmARG numeration) shows a different conformation in LmARG. Finally, differences in the intermolecular interactions that stabilize the homotrimer were found [15].

On the other hand, arginase-knockout mutants from *L. mexicana*, *L. amazonensis* and *L. major* demonstrated the importance of this enzyme since its absence made parasites auxotrophic to polyamines [13,16,17]. Actually, there are several arginase inhibitors reported in different *Leishmania* species, in the case of *L. amazonensis* (LaARG), [1,2,4]triazolo [1,5-a]pyrimidine derivatives [17], flavanols [18,19], and cinnamide derivatives [20] had been reported, with the best compounds showing a mixed type inhibition and a I_50_ in the micromolar range. In *L. infantum* (LiARG), natural phenolic derivatives [21] and chalcone derivatives [22] have been reported as enzyme inhibitors. In LmARG, substrate or transition state analogues inhibited the enzyme [23,24]. Here, the most potent compounds (ABH, BEC and NOHA) inhibited LmARG in a competitive way, with Ki values of 1.3–85 µM, however, they were not selective to LmARG [23]. Furthermore, α,α-disubstituted boronic amino acid inhibitors were capable to inhibit LmARG showing a special type of binding [24].

In this work, we applied a virtual screening strategy from our *in-house* benzimidazole derivatives chemical library to find new and selective LmARG inhibitors. Kinetic and molecular dynamics characterization studies were performed, as well as their biological activity against the two stages of the parasite and their cytotoxic effect in murine macrophages. Moreover, its selectivity against HsARG and a prediction of their ADME-Tox properties were also made.

## 2. Results

### 2.1. Virtual Screening and LmARG Inhibition

With the aim to find new LmARG inhibitors that recognize the active site of the enzyme, we performed a virtual screening, using molecular docking, of our *in-house* library of 450 benzimidazole derivatives using the crystal structure of LmARG (PDB ID: 4IU0). The ligands were ranked according to their respective docking scores.

The fifty molecules with the highest score were chosen for inhibition studies. According to data, 33 compounds inhibited LmARG in a range of 8–100% at a concentration of 200 μM (Table S1). The ten compounds that induced the highest LmARG inhibition are shown in Table 1. As can be observed, compounds **1** and **2** are the most effective against the enzyme at the evaluated concentration. Nonetheless, some interesting structural activity relationships can be analyzed from these data. The changing of a methylcarbamate in position 2 of the benzimidazole nucleus by an acetyl group, reduce almost 50% the inhibitory potency (compound **1** vs. **4**). Additionally, only changing the chloride from position 5 to 6 diminishes the inhibition 60% (compound **1** vs. **8**). In the same context, substituting the chloride by a larger and hydrophobic group such as propylthio, has the same effect (compound **1** vs. **9**). On the other hand, the change of carboxamide in position 5 by a piperazine group provokes more than 50% lost in potency (compound **2** vs. **6**). Additionally, when the size of the R group attached to sulfur, at position 2, is larger and with a greater degree of freedom, the activity decreases 40 or 60% in comparison with a small group (**2** vs. **3** and **7**).

**Figure 1:**
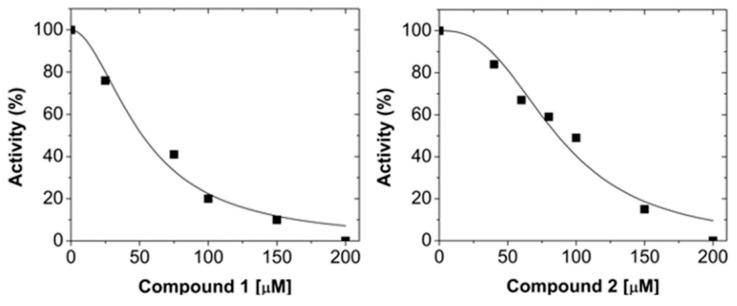
Inhibition kinetics of recombinant LmARG by compounds **1** and **2**.

To continue with kinetic characterization, the concentration that inhibits 50% enzyme activity (I_50_) was determined for the two most potent compounds (**1** and **2**). The data show that compounds **1** and **2** have an I_50_ value of 52 μM and 82 μM, respectively (Figure 1). Both compounds show a cooperative behavior, this fact suggests that more than one molecule are required to inhibit the enzyme [25,26].

### 2.2. Human Arginase 1 Inhibition

**Figure 2:**
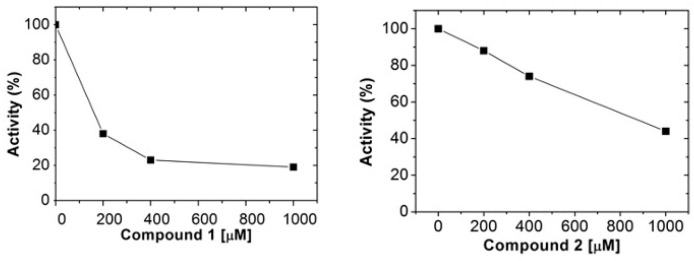
Inhibition kinetics of recombinant HsARG by compounds **1** and **2**.

Nowadays, one of the principal concerns in LmARG inhibitors design is their selectivity against human arginase 1 (HsARG) [23,27]. Therefore, the potential inhibitory activity of both compounds against recombinant HsARG was determined. The results show that at the highest concentration possible to be tested (1 mM) none of the compounds inhibit completely the enzyme (Figure 2), whilst 100% inhibition of recombinant LmARG was achieved when compounds **1** and **2** were used five times less concentrated (Table 1 and Figure 1). Therefore, this data demonstrate that compounds **1** and **2** are selective to the enzyme from parasite. As far as we know, this is the first study that reports LmARG selective inhibitors.

### 2.3. Molecular Dynamics

**Figure 3:**
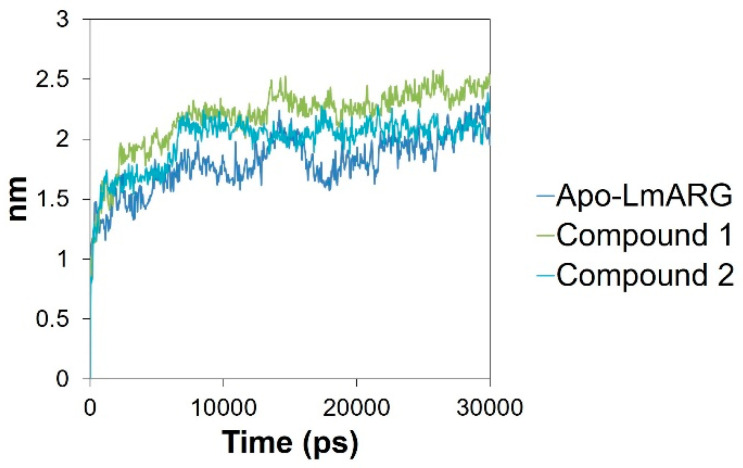
Root Mean Square Deviation analysis of Cα in the Apo-LmARG and the respective complexes.

Once the kinetic studies were caried out, it was important to know how these inhibitors interact with the enzyme. To investigate the binding mode of both compounds in LmARG, molecular dynamics simulations of 30 ns were performed. First, to explore the dynamic stability of systems, Root Mean Square Deviation (RMSD) was analyzed in Apo-LmARG and the complexes (Figure 3). The observed deviation through the simulation time was 0.15 nm approximately, which indicates that the systems were stable throughout the simulation.

**Figure 4:**
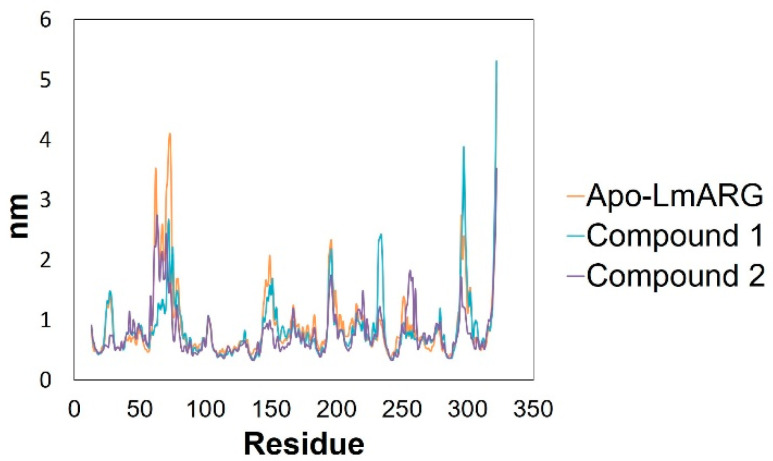
Root Mean Square Fluctuation analysis of Cα in the Apo-LmARG and the respective complexes.

Subsequently, the Root Mean-Square Fluctuation (RMSF) between Apo-LmARG, and each LmARG-compound complex was analyzed to visualize local changes along the protein chain (Figure 4). Large variations in some regions were observed, therefore, an ANOVA analysis was realized between Apo-LmARG and both complexes, getting p values of 0.0001 (*p* < 0.05 significative) in both cases. These results indicate that the binding of the compounds cause the fluctuations observed.

**Figure 5:**
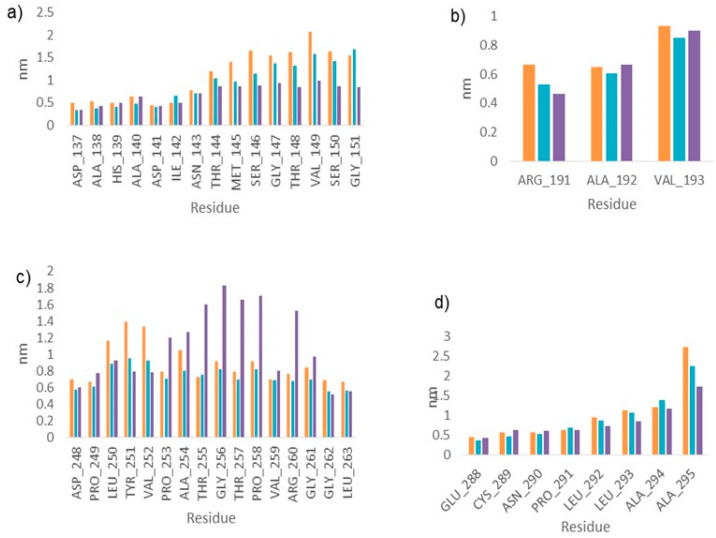
Root Mean Square Fluctuation analysis of Cα in (**a**) loop 1, (**b**) loop 2, (**c**) loop 3, and (**d**) loop 4. Apo-LmARG (orange), LmARG-compound **1** (blue), LmARG-compound **2** (violet).

The regions with the highest variations correspond to diverse protein loops. Arginases possess various important loops that participate in the opening of the substrate binding site or enclosing the active site cavity. Therefore, an analysis of the residues that forms those loops in Apo-LmARG and the two complexes was performed. The data show that in both complexes, the loops that participate in the binding of arginine carboxylic acid, as well as the primary amine binding region, were the loops that show less flexibility in the Cα fluctuations (Figure 5). This behavior was like that reported by Mortier et al. 2017, where it was observed, in the comparison of Cα in the RMSF, that there were larger movements with Apo-LmARG than with arginase-ligand complexes, indicating that the arginase binding site is a highly flexible site [27].

### 2.4. LmARG-Inhibitor Interactions

**Figure 6:**
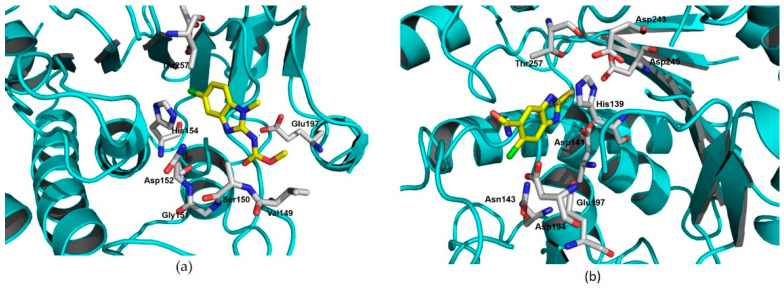
Ligand interactions in LmARG-inhibitor complexes after 30 ns of MD simulation. (**a**) compound **1** and (**b**) Compound **2**. Both inhibitors are represented as yellow sticks for the C atoms. Relevant residues surrounding the inhibitor are depicted as capped sticks and labeled. Predicted polar interactions (Glu197 and Asp141, respectively) depicted dashed lines.

In order to understand how these inhibitors behaved along MD simulation, a detailed analysis of their interactions was carried out. Compound **1** interacts only in the first 10 ns through a hydrogen bound with Asn143, and at the end of the simulation makes another hydrogen bound with Glu 197. Moreover, there are also Van der Waals interactions with His139, Ala140, Val149, His154, Ala192, Val193, Ile200, Met211, Val259, Leu263, and ionic interactions with Asp137, Asp141, Asp243, Asp245 and His139 (Figure 6a).

On the other hand, compound **2** forms a pi-pi interaction in the first 10 ns between the His154 and the benzimidazole ring, in addition to a hydrogen bond interaction between the Asp141 and the NH of the benzimidazole ring, interestingly, it was maintained during all simulation time. Moreover, there are Van der Waals interactions with His139 and His154, and ionic interactions with His114, Asp137, His139, Asp141, Asp243, Asp245, and Glu288 (Figure 6b).

### 2.5. Biological Activity Evaluation and Cytotoxicity

To continue the characterization of these inhibitors, an important issue was to investigate their biological activity. Therefore, they were tested for antileishmanial activity against promastigotes and amastigotes from *L. mexicana*. The results show that both compounds have leishmanicidal activity in the promastigote and in the intracellular amastigote. Compound **1** showed to be more active in both promastigote (IC_50_ = 64 µM) and amastigote (IC_50_ = 32 µM) than the Glucantime, nonetheless, it was less active than Amphotericin B and Miltefosine (Table 2). Regarding to cytotoxicity, both compounds have CC_50_ values >417 µM against murine macrophages J774.2 cell line and a selectivity index (SI) higher than the reference drugs Glucantime, Amphotericin B and Miltefosione. (Table 2).

### 2.6. In Silico Physichemical and ADME-Tox Predictions

In the first steps of drug design process the Drug-like properties of the studied molecules is an issue of interest. With this in mind, the physicochemical parameters and ADME-Tox properties of compounds **1** and **2** were predicted through an in silico study as described in methodology. The results show that both compounds fulfilled the Lipinski, Veber and Egan rules, suggesting that these molecules have a good oral biodisponibility (Table 3). Moreover, toxicity studies predict that any of these compounds should bind to proteins that have been associated with adverse reactions and toxic effects (Table 3). The oral toxicity prediction shows that compounds **1** and **2** would have a LD_50_ of 5050 mg/kg and 750 mg/kg, respectively. Furthermore, compound **1** belongs to the toxicity class 6 which indicates that should be safe to use, while compound **2** belongs to the toxicity class 4 which indicates that it could be harmful but only if >2000 mg/kg have been administered. Therefore, both molecules possess Drug-like properties and are likely to have good absorption and eventually do not show any toxic effects.

## 3. Discussion

This study describes the effect of two benzimidazole derivatives as selective LmArg inhibitors and leishmanicidal agents. These compounds have a common benzimidazole nucleus present in several approved drugs, most of them substituted at position 2 [29] as shown in compounds **1** and **2**. Other 2-substituted benzimidazole derivatives have also shown activity as analgesics, anti-inflammatory [30] and antibacterial agents [31]. Furthermore, docking studies showed that 2, 5 (6) substituted benzimidazole derivatives are also molecules with potential as antimicrobial agents [32]. The importance of the substituents in position 2 and 5 in benzimidazole had been also reported [33]. All of the above highlights that these positions are promising for the development of new drugs.

Until now there are few studies about LmARG inhibitors, these compounds are product or transition states analogs, which do not confer selectivity towards the parasite enzyme [24]. On the other hand, in α,α-disubstituted boronic amino acid derivatives were suggested to maintain enzyme affinity while adjusting the intermolecular interactions of the δ chain, however experimental data are not yet available [24]. Nieto et al., 2018, demonstrated the leishmanicidal activity of one benzimidazole derivative, named compound **8**, against the promastigotes and amastigotes of *L. mexicana* (IC_50_ = 3.25 and 0.26 µM, respectively). In addition, authors evaluated the inhibitory activity of compound **8** against recombinant LmARG using the Quantichom Arginase Activity Assay kit, and determined an I_50_ of 35.9 µM. Nevertheless, the difference between I_50_ and IC_50_ values, many times lower in the parasite, suggested that there are more targets for these molecules in the parasite apart from arginase [34]. To the contrary, this relationship (I_50_ and IC_50_ values) in compounds **1** and **2** suggest that arginase could be the principal target in the parasite (Figure 1 and Table 2). Therefore, the strategy of target directed drug design was helpful to obtain molecules with, in principle, more specificity.

Interactions observed in LmARG-inhibitor complex with Asp141, Asn143, and Glu197 have been reported in other LmARG inhibitors, such as nor-NOHA, 2-(S)-amino-6-boronohexanoic acid (ABH), and its derivatives (R)-2-amino-6-borono-2-[2-(piperidin-1-yl)ethyl]hexanoic acid (ABHPE) and (R)-2-amino-6-borono-2-[1-(3,4 dichlorobenzyl)piperidin-4-yl]-hexanoic acid (ABHDP) [15,24]. In particular, the interaction with Asp141 was also found in complexes of LaARG with quercetin, quercitrin and isoquercitrin, where the two hydroxyls in catechol group of the flavonoids formed a hydrogen bond with Asp141 [18]. Moreover, in a study of cinnamide inhibitors evaluated in LaARG, it was observed that some of these inhibitors share Van der Waals interactions with the same residues as compound **1** and **2** (His139, Val149, and His154), as well as electrostatic interactions with Asp245 and Glu288 [20]. Furthermore, several of these Van der Waals interactions were also found with chalcone inhibitors in *L. infantum* [22].

On the other hand, biological activity and cytotoxicity studies indicated that both LmARG inhibitors had leishmanicidal activity being compound **1** as active as the reference drug Glucantime (32 and 35 µM, respectively) and more selective against the amastigote stage of the parasite than the three reference drugs used for the treatment of leishmaniasis (Table 3). In the same context, several cinnamides derivatives were reported as LaARG inhibitors and tested against *L. amazonensis* promastigotes, but only caffeic acid phenylamide (CAPA) showed biological activity with an IC_50_ of 82.56 µM, however, no data over its effect against mammalian cell lines are available [20].

Compounds of synthetic origin such as benzophenone [35] and 5-nitroindazole derivatives have also been reported with leishmanicidal activity against *L. infantum*, *L. braziliensis*, and *L. donovani* and their cytotoxic effect on macrophages was also evaluated [36]. These compounds showed biological activity in the micromolar order and also exhibited selectivity, similar results as those reported in our study. Additionally, several LmARG inhibitors have been reported, nonetheless, assays in parasites only have been done with NOHA and nor-NOHA inhibitors, which showed an EC_50_ in the millimolar order in promastigotes [23], in comparison with compounds **1** and **2** that act in the micromolar range (Table 2). It is of interest to evaluate in future studies the leishmanicidal activity of compounds **1** and **2** against other *Leishmania* spp. causative of the other clinical manifestations of leishmaniasis. Altogether, the results reported in our study suggest that the two benzimidazole derivatives are an excellent starting point for design new drugs against leishmanisis.

## 4. Materials and Methods

### 4.1. Virtual Screening

The crystal structure of LmARG in complex with inhibitor ABH (PDB ID: 4IU0) [15] was selected from the Protein Data Bank. The protein was prepared using Protein Preparation Wizard tool [37] (Schrödinger Release 2016: Glide, Schrödinger, LLC, New York, NY, USA). All unnecessary ligands and the water molecules were removed except the Mn^2+^ ions, which are important in enzymatic catalysis. Furthermore, in this module bond orders were assigned, hydrogens were added, and zero-order bonds to metals were created. The structure was minimized with the OPLS2005 forcefield [38,39].

Our *in-house* chemical library conformed by 450 benzimidazole derivatives was used for molecular docking virtual screening. Compounds were prepared using the module LigPrep (Schrödinger Release 2016: LigPrep, Schrödinger, LLC, New York, NY, USA) to generate low-energy 3D structures with ionization states of pH 7 +/− 0.5 using OPLS2005 forcefield. The docking was made on the active site selecting the manganese atoms of the LmARG as center, with a grid box size of 10 × 10 × 10. Docking studies were performed using Glide in its three modes, Standard Precision, Extra Precision, and Induced Fit Docking (IFD) using default parameters [40,41,42]. The complexes obtained from IFD were used for molecular dynamics simulation studies.

### 4.2. Cloning of L. mexicana Arginase Gene

The gen *ARG* that encodes for arginase from *L. mexicana* (LmARG) was amplified using the following oligonucleotides 5′-GCCATATGGAGCACGTGCAGCAGTACAAGTTCTAC-3′ (forward) and 5′-GC-GGATCCCTATAGCTTGGAGCTCGTATGCGGAGTGTA-3′ (reverse). Restriction sites for *NdeI* and *BamHI* were introduced in the forward and reverse oligonucleotides, respectively. The PCR product was cloned using PCR BLUNT II^®^ TOPO^®^ vector (Invitrogen, Carlsbad, CA, USA), and then was subcloned into pET28a(+). The resulting plasmid was transformed in *E. coli* BL21(DE3)pLysS competent cells (Novagen, Madison, WI, USA).

### 4.3. Cloning of Human Arginase 1 Gene

The gen *ARG1* that encodes for arginase 1 from human (HsARG) was amplified from HepG2 cells RNA [HEPG2] (ATCC^®^ HB-8065™) by RT-PCR using oligonucleotides 5′GCCATATGAGCGCCAAGTCCAG-3′ (forward) and 5′-GGGTGGATTCATTCCTAGGCG-3′ (reverse). Restriction sites for *NdeI* and *BamHI* were introduced in the forward and reverse oligonucleotides, respectively. The PCR product was cloned into pJET^®^ plasmid and then was subcloned into pETHISTEV [43]. The resulting vector was transformed in *E. coli* BL21(DE3)pLysS competent cells (Novagen, Madison, WI, USA).

### 4.4. Expression and Purification of Arginases

Bacteria transformed with recombinant plasmid were grown in Luria-Bertani medium containing 50 µg/mL kanamycin (LmARG) or 50 µg/mL ampicillin (HsARG1) at 37 °C. Once the cultures reached an OD_600_ nm of 0.8, isopropyl β-D-thiogalactopyranoside (IPTG) was added (1 mM) to induce overexpression and incubated overnight. Thereafter, cells were harvested by centrifugation at 5000 rpm during 30 min. The cells were resuspended in buffer A (50 mM potassium phosphate (pH 7.0), 300 mM NaCl, 10% (*v*/*v*) glycerol) supplemented with the protease inhibitor PMSF (200 µM) and then lysed by sonication. The lysate was centrifuged at 12,000 rpm for 30 min. LmARG and HsARG were purified through affinity chromatography, the supernatant was loaded into a column with Ni-NTA resin pre-equilibrated in buffer A. The column was washed with buffer A supplemented with an imidazole gradient of 50, 150, and 300 mM. Both enzymes eluted in imidazole 300 mM. Protein concentrations were determined by the Bradford method [44].

### 4.5. Enzyme Activity

The activity of the recombinant arginases was measured in a colorimetric assay with some modifications [45]. Briefly, the enzyme (2 µg) was previously activated with 1 mM MnCl_2_, 100 mM Tris-HCl, pH 8 during 15 min at 37 °C. Thereafter, 50 mM L-arginine was added, and the reaction was stopped after 10 min using 30 µL of an acid mixture (H_2_SO_4_, H_3_PO_4_ and H_2_O [1:3:7]), and 20 µL of 245 mM of α-isonitrosopropiophenone (dissolved in ethanol). Samples were heated in a thermocycler at 95 °C for 1 h. The urea in the samples was measured at 550 nm in a Synergy H1 Hybrid Multi-Mode Microplate Reader (BioTek).

### 4.6. Inhibition Assays

The fifty molecules with the highest docking score were selected for inhibition studies that were made under the conditions mentioned above, adding the respective compound (dissolved in DMSO) at a concentration of 200 µM, keeping a final 10% DMSO concentration. The concentration that inhibits 50% of the enzyme activity (I_50_) was determined through curves at different compound concentrations. The I_50_ was obtained fitting the curves to the equation reported by Téllez-Valencia, et al., 2002 [25].

### 4.7. Molecular Dynamics

Molecular dynamics simulations with the following systems, Apo-LmARG, LmARG-compound-**1**, and LmARG-compound-**2** were performed using Desmond software [46] (Schrödinger Release 2016: Desmond Molecular Dynamics System, D. E. Shaw Research, New York, NY, 2016. Maestro-Desmond Interoperability Tools, Schrödinger, New York, NY, USA). The complexes were centered in an orthorhombic box and filed with an explicit solvent using the TIP4PEW model of water. The systems were neutralized with counter ions of Na^+^ to balance the net charges of the systems. Minimization and equilibration were simulated using the default parameters. The simulation was performed during 30 ns and the data were collected every 100 ps. The pressure was maintained at 1 atmosphere and temperature at 300 K, using an NPT ensemble.

### 4.8. In Silico ADME-Tox Prediction

FAFDrugs4 [47] and ADMET-SAR [48] servers were used to predict ADME-Tox properties of compounds whilst ProTox [49] server was used for prediction of toxicities. Some of descriptors determined were molecular weight (MW), logarithm of the partition coefficient n-octanol/water (logP), octanol water distribution coefficient (logD), intrinsic water solubility (logSw), topological polar surface area (tPSA), rotable bonds (RB), hydrogen bond donors (HBD), hydrogen bond acceptors (HBA), Lipinski´s rules violations, Veber Rules, Egan Rules, Predicted LD50, Predicted toxicity class, and Toxicity targets.

### 4.9. Biological Activity against Leishmania mexicana Promastigotes

Promastigotes of *L. mexicana* (MNYC/BZ/62/M379 reference strain) were grown in Schneider’s medium supplemented with fetal bovine serum 10% (*v*/*v*), ampicillin (100 U/mL)/streptomycin (100 µg/mL). Parasites were collected at log phase to assess the compounds and 5 × 10^5^ promastigotes were seeded in a 96-wells plate. Compounds were previously dissolved in dimethyl sulfoxide (DMSO) up to a concentration of 1 mg/mL. Thereafter, serial dilutions were made with culture medium from 100 µg/mL to 0.8 µg/mL and then they were added to the wells by triplicates. Parasites non treated and treated with Glucantime, Amphotericin B and Miltefosine were included as positive and negative controls, respectively. Parasites were incubated at 26 °C during 48 h and resazurin 2.5 mM was added at the end into each well. Afterwards, fluorescence intensity was measured with excitation/emission 535/590 nm in a spectrofluorometer (Infinite 200, Tecan Group Ltd., Mannedorf, Switzerland). Finally, half maximal inhibitory concentration (IC_50_) was determined through a Probit analysis [50]. The assays were made by triplicate in 3 independent experiments.

### 4.10. Biological Activity against Leishmania mexicana Amastigotes

Biological activity of the benzimidazole derivatives was also evaluated against the amastigote of *L. mexicana.* For this, 5 × 10^4^ murine macrophages J774.2 (ATCC^®^ TIB-67) per well were grown in a plate of 96 wells with RPMI medium at 33 °C, 5% CO_2_ during 24 h. The cells were infected with promastigotes in log phase in a 10:1 proportion (parasite: macrophage) and they were incubated at 37 °C, 5% CO_2_ during 24–48 h. A wash with RPMI medium to remove the not-infected promastigotes was made. Compounds were added in a serial dilution from 100 µg to 0.8 µg/mL in triplicate and were incubated at 37 °C, 5% CO_2_ for 48 h. Glucantime, Amphotericin B and Miltefosine were used as reference drugs. Then, the cells were lysed with a non-complemented Schneider’s medium lysis solution with SDS 0.01%, after incubating for 20 min the lysis solution was removed. Afterwards, Schneider medium was added incubating at 26 °C during four days at room temperature to differentiate intracellular amastigotes from free amastigotes. Thereafter, 20 µL of resazurin 2.5 mM was added to each well and incubated during 3 h. Fluorescence intensity and IC_50_ were measured as described for promastigotes. The assays were made by triplicate in 3 independent experiments.

### 4.11. Cytotoxicity Assays

Murine macrophages J774.2 (ATCC^®^ TIB-67) (5 × 10^4^ cells/well) were grown in plates of 96 wells for 24 h at 37 °C in 5% CO_2_. Thereafter, compounds were added in serial diluted concentrations (100 to 0.8 µg/mL) and they were incubated during 24 h. The cellular vitality was evaluated using resazurin, adding 20 µL to each well at 2.5 mM incubating 4 h. Finally, the cytotoxic concentration (CC_50_) was determined through a Probit analysis [50]. The assays were made by triplicate in 3 independent experiments. CC_50_ values of Amphotericin B, Miltefosine and Glucantime were taken from a previous study [28] performed under the same conditions by our research group.

### 4.12. Synthesis of Benzimidazole Derivatives

**Scheme 1:**
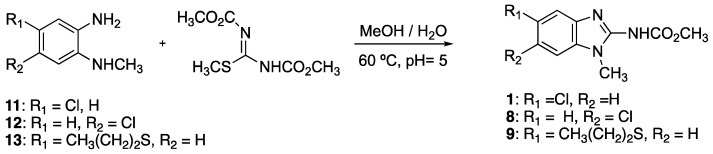
Synthesis of compounds **1**, **8**, **9**.

Compounds **1**, **8**, and **9** were prepared from the properly substituted o-phenylenediamines, according to methods reported for our group [51,52,53]. Briefly, compounds **11**–**13** were treated with 1.2 equivalents of 1,3-dicarbomethoxy-S-methylisothiourea in MeOH/H_2_O at pH 5 at reflux point to give a solid which was recrystallized from methanol with activated charcoal. Scheme 1.

Methyl (5-chloro-1-methyl-1*H*-benzimidazol-2-yl)carbamate (**1**). Yield 85%. Recrystallized from ethanol with charcoal. mp. 203.0–203.8 °C. ^1^H NMR (Acetone-*d*_6_; 300 MHz) δ: 3.04 (s, 3H, NCH_3_), 3.17 (s, 3H, OCH_3_), 6.77 (dd,1H, *J* = 8.4 Hz, *J* = 1.8 Hz, H-6), 6.94–6.98 (m, 2H, H-4, H-7), 11.65 (s, 1H, NH). MS MS (EI^+^): *m/z* 239 [M]^+^.

Methyl (6-chloro-1-methyl-1*H*-benzimidazol-2-yl)carbamate (**8**). Yield 80%. Recrystallized from ethanol/H_2_O with charcoal. mp. 191.3–192.4 °C. ^1^H NMR (CDCl_3_; 300 MHz) δ: 3.72 (s, 3H, NCH_3_), 3.82 (s, 3H, OCH_3_), 7.25–7.34 (m, 3H, H-4, H-5, H-7), 9.91 (d, 1H, NH). MS (EI^+^): *m/z* 239 [M]^+^.

Methyl (1-methyl-5-(propylthio)-1*H*-benzimidazol-2-yl)carbamate (**9**). Yield 80%. Recrystallized from methanol with charcoal. mp. 120.5–122.1 °C. ^1^H NMR (DMSO-*d*_6_; 300 MHz) δ: 0.93 (t, 3H, *J* = 7.5 Hz, CH_3_CH_2_CH_2_S-), 1.53 (m, *J* = 7.5 Hz, 2H, CH_3_CH_2_CH_2_S-), 2.85 (t, 2H, *J* = 6.9 Hz, CH_3_CH_2_CH_2_S-), 3.46 (s, 3H, NCH_3_), 3.60 (s, 3H. OCH_3_), 7.19 (dd, 1H, *J* = 8.4 Hz, *J* = 1.5 Hz, H-6), 7.32 (d, 1H, *J* = 8.1 Hz, H-7), 7.42 (s, 1H, H-1), 11.98 (s, 1H, NH). ^13^C NMR (DMSO-*d*_6_; 75 MHz) δ: 13.01, 21.99, 28.03, 36.16,51.72, 109.64, 112.78, 124.35, 128.71, 129.21, 129.59,153.5, 162.51. MS (FAB^+^): *m/z* 280 [M^+^H] ^+^.

**Scheme 2:**
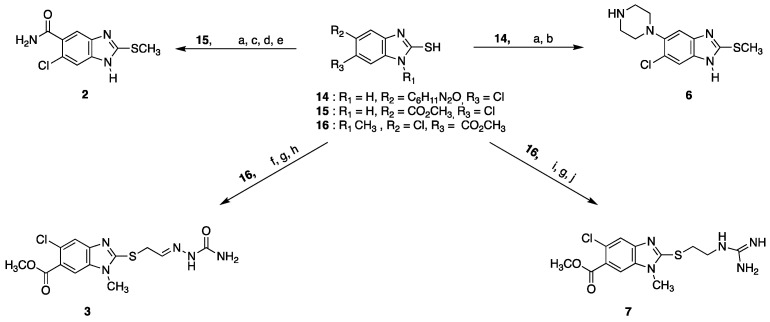
Synthesis of compound 2, 3, 6 and 7. Reactants and conditions: (a) CH_3_I/KOH, acetone, 0 °C; (b) EtOH/H_2_O, H_2_SO_4_ cat., 50 °C; (c) KOH, MeOH, 65 °C; (d) SOCl_2_, DMF, 50 °C; (e) NH_3_, NH_4_OH, MeCN, rt., 24 h; (f) bromoacetaldehyde diethyl acetal, K_2_CO_3_, DMF, rt.; (g) HCl _2_N, 1,4-dioxane, rt.; (h) semicarbazide hydrochloride, EtOH, NaHCO_3_, rt., 2 h; (i) tert-butyl (2-bromoethyl)carbamate, acetone, K_2_CO_3_, rt.; (j) 1H-pyrazole-1-carboxamidine hydrochloride, TEA, MeCN, 60 °C, 24 h.

Compounds **2**, **3**, **6**, and **7** were prepared from the properly substituted 1*H*-benzimidazole-2-thioles **14**, **15**, and **16**, which were synthesized according to procedures reported by our group [52,54,55,56]. The synthetic methodology is resumed in Scheme 2.

Compound **2** was obtained from **15** by a series of four steps: *S*-methylation with methyl iodide, basic hydrolysis of the ester group, acyl chloride preparation with SOCl_2_ and DMF in catalytic quantities, and finally, the carboxamide synthesis by treatment with ammonia freshly generated in acetonitrile at room temperature [55].

Compound **6** was obtained from **14** by an *S*-methylation reaction with methyl iodide, according to our reports, and subsequent acid hydrolysis of the acetyl protecting group [52,56].

Compound **3** was obtained from **16**, initially, this was converted in the 2-((2-oxoethyl)thio)derivative by reaction with bromoacetaldehyde diethyl acetal followed by acid hydrolysis (HCl in dioxane); then, the aldehyde was reacted with semicarbazide hydrochloride in ethanol and a base at room temperature [57]. The product was purified by recrystallization.

Compound **7** was prepared by a sequence of three reactions: preparation of 2-((2-((*tert*-butoxycarbonyl)amino)ethyl)thio derivative by treatment **16** with *tert*-butyl (2-bromoethyl)carbamate in acetone in a basic medium; then, the Boc protecting group was removed by hydrolysis with HCl in dioxane; finally, the free amine was treated with 1*H*-pyrazole-1-carboxamidine hydrochloride in acetonitrile and triethylamine at 60 °C to give 7 [58].

6-Chloro-2-(methylthio)-1*H*-benzimidazole-5-carboxamide (**2**) Yield 21%, column chromatography (Al_2_O_3_, EtOH:MeOH, 1:1). mp 238.8–240.4 °C. ^1^H NMR (DMSO-*d*_6_; 400 MHz) δ: 2.7 (s, 3H, SCH_3_), 7.47(s, 1H, CONH_2_), 7.49 (s, 1H, H-4), 7.51 (s, 1H, H-7), 7.78 (s, 1H, CONH_2_), 12.83 (s, 1H, NH). ^13^C NMR (DMSO-*d*_6_; 100 MHz) δ: 13.8, 122.9, 130.1, 154.4, 168.7. MS (DART^+^) *m/z*: 242 [M+H]^+^. HRMS (DART^+^) cal. [C_9_H_8_ClN_3_OS +H] 242.01549, found 242.01566

6-Chloro-2-(methylthio)-5-(piperazin-1-yl)-1*H*-benzimidazole (**6**). Yield 42%. Purified by precipitation from ethanol/HCl gas. mp 235–237 °C. ^1^H NMR (DMSO-*d*_6_; 300 MHz) δ: 2.50 (s, 3H, SCH_3_), 3.17–3.25 (m, 8H, piperazine), 7.32 (s, 1H, H-4), 7.69 (s, 1H, H-7), 9.42 (b, 2H, NH). ^13^C NMR (DMSO-*d*_6_; 75 MHz) δ: 14.57, 43.13, 48.47, 105.09, 114.73, 124.33, 132.23, 134.64, 144.45, 153.10. MS (EI^+^) *m/z*: 282 [M]^+^.

Methyl 2-((2-(2-carbamoylhydrazineylidene)ethyl)thio)-5-chloro-1-methyl-1*H*-benzimidazole-6-carboxylate (**3**). Overall yield 8.37% Recrystallized from isopropyl alcohol. mp 196.7–197.1 °C. ^1^H NMR (DMSO-*d*_6_; 300 MHz) δ: 3.72 (s, H, NCH_3_), 3.86 (s, 3H, CH_3_O), 4.13 (d, 2H, *J* = 4.7 Hz, SCH_2_CHN), 6.21 (sb, NH), 7.34 (t, 1H, 4.6, SCH_2_CHN),7.81 (s, 1H, H-4), 7.98 (s, 1H, H-7), 10.07 (b, 1H, NH). ^13^C NMR (DMSO-*d*_6_; 75 MHz) δ: 30.76, 33.7, 52.78, 112.30, 120.32, 123.27, 125.65, 137.49, 139.86, 141.33, 154.95, 156.80, 166.15. MS (ESI^+^): *m/z* 378 [M^+^Na]^+^.

Methyl 5-chloro-2-((2-guanidinoethyl)thio)-1-methyl-1*H*-benzimidazole-6-carboxylate (**7**). Overall yield 37%. Recrystallized from methanol. mp 172.9–173.4 °C. ^1^H NMR (DMSO-*d*_6_; 400 MHz) δ: 3.50–3.44 (m, 4H, SCH_2_CH_2_NH), 3.69 (s, 3H, NCH_3_), 3.85 (s, 3H, CH_3_O), 7.79 (s, 1H, H-4), 7.29 (sb, 2H, NH_2_), 7.54 (sb,1H, NH), 7.98 (s, 1H, H-7), 8.19 (sb, 1H, NH). ^13^C NMR (DMSO-*d*_6_; 100 MHz) δ: 30.4, 30.8, 40.5, 52.3, 112.2, 120.4, 123.2, 125.8, 139.0, 141.2, 155.7, 157.5, 166.4. MS (ESI^+^): *m/z* 342 [M+H]^+^.

On the other hand, the synthesis of compounds **4**, **5**, and **10** have already been reported by our group [59].

### 4.13. Statistical Analysis

Analysis of the MD data, specifically RMSF, was carried out by an ANOVA test using SPSS v15.0 software. Differences between data were considered statistically significant when a *p*-value < 0.05.

## 5. Conclusions

Two novel benzimidazole derivatives (compounds **1** and **2**) were discovered through virtual screening as the first selective LmARG inhibitors. Moreover, showed activity against the promastigote and amastigote stage of *L. mexicana* in the micromolar range. Compound **1** showed a higher Selectivity Index against the parasite amastigote than the reference drugs. Furthermore, the in silico prediction of their ADME-Tox properties suggested that both inhibitors supports the structural characteristics to be considered drug candidates. Therefore, the compounds reported here provides a starting point to develop more powerful and selective LmARG inhibitors to continue in the search of new drugs against leishmaniasis.

## Figures and Tables

**Table 1:** Benzimidazole derivatives with the highest inhibition of recombinant LmARG.

Compound	Chemical Structure	Docking Score (kcal/mol)	% Inhibition LmARG [200 μM]
**1**	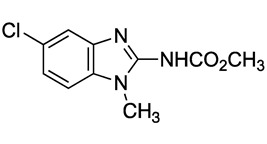	**−5.46**	**100%**
**2**	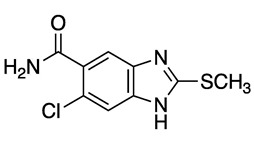	**−5.04**	**100%**
**3**	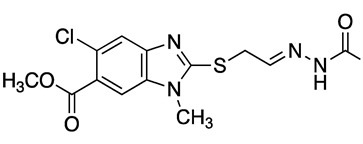	**−6.39**	**60%**
**4**	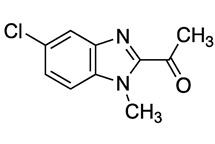	**−6.05**	**56%**
**5**	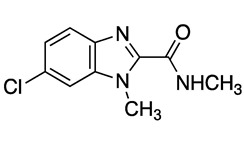	**−5.96**	**56%**
**6**	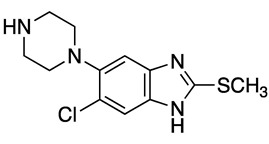	**−8.38**	**46%**
**7**	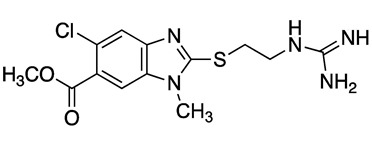	**−6.05**	**40%**
**8**	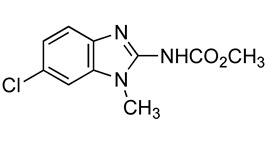	**−6.00**	**40%**
**9**	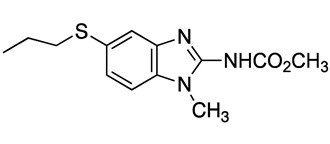	**−5.04**	**36%**
**10**	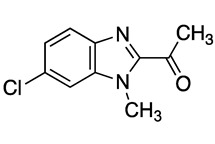	**−6.65**	**33%**

**Table 2:** Leishmanicidal activities, cytotoxicity and selective index of compounds **1**, **2** and reference drugs.

Compound	IC_50_ (µM)		CC_50_ (µM)	SI ^1^	
	Promastigotes	Amastigotes		Promastigotes	Amastigotes
**1**	64	32	>417 ^2^	6.5	13
**2**	132	101	>414 ^2^	3.1	4.1
Glucantime	101	35	>273 ^3^	2.7	7.8
Amphotericin B	0.89	1.3	7.4 ^3^	8.3	6.2
Miltefosine	8.1	8.6	141 ^3^	17.4	2

^1^ The selectivity index (SI) was calculated as the ratio of cytotoxicity to biological activity (SI = CC_50_/IC_50_). ^2^ The results show the maximum concentration evaluated in the experiments. ^3^ Data obtained from Matadamas-Martínez et al., 2019 [28].

**Table 3:** In-silico predicted physicochemical parameters and ADME-Tox profiling of compounds **1** and **2**.

Descriptor	Compound 1	Compound 2
MW ^1^	239.66	241.7
logP ^1^	2.35	2.1
logD ^1^	2.63	2.03
logSw ^1^	−2.93	−2.82
tPSA ^1^	56.15	97.07
RB ^1^	2	2
HBD ^1^	1	3
HBA ^1^	5	4
Lipinski’s violations ^1^	none	none
Veber Rules ^1^	Good	Good
Egan Rules ^1^	Good	Good
BBB+ ^2^	0.9584	0.9117
HIA+ ^2^	0.9965	0.9819
Caco2 ^2^	0.6531	0.5468
Lysosome ^2^	0.6217	0.6217
Predicted LD_50_^3^	5050 mg/kg	705 mg/kg
Predicted toxicity class ^3^	6	4
Toxicity targets ^3^	no binding	no binding

^1^ Obtained in FAFDrugs4 server; MW, Molecular weight MW. LogP, logarithm of the partition coefficient n-octanol/water; logD octanol water distribution coefficient; logSw, intrinsic water solubility; tPSA, topological polar surface area; RB, rotable bonds; HBD, hydrogen bond donors; HBA, hydrogen bond acceptors. ^2^ Obtained in ADMET-SAR server; BBB+, Blood-Brain Barrier; HIA+, Human Intestinal Absorption; Caco2, Caco2 Permeability. ^3^ Obtained in ProTox server; Predicted toxicity class values ranged between 1 to 6, 1 is toxic and 6 is safe. Toxicity targets were determined for: Adenosine A2A receptor, Adrenergic beta 2 receptor, Androgen receptor, Amine oxidase, Dopamine D3 receptor, Estrogen receptor 1 and 2, Glucocorticoid receptor, Histamine H1 receptor, Nuclear receptor subfamily 1 group I member 2, Opioid receptor kappa, Opioid receptor mu, cAMP-specific 3 5 -cyclic phosphodiesterase 4D, Prostaglandin G/H synthase 1, Progesterone receptor.

## Supplementary Materials

The following are available online at https://www.mdpi.com/article/10.3390/ijms222413613/s1.
