# Supplementary material for: Benzimidazole Derivatives as New and Selective Inhibitors of Arginase from Leishmania mexicana with Biological Activity against Promastigotes and Amastigotes
Source: Int J Mol Sci. 2021 Dec 19;22(24):13613. doi: 10.3390/ijms222413613 (PMC8705706; doi:10.3390/ijms222413613)
Supplement: Supplementary file 1 [file ijms-22-13613-s001.zip › ijms-1512343-supplementary.pdf]

Table S1. Inhibition of recombinant LmARG by benzimidazole derivatives.

Compound	Chemical structure	% Inhibition LmARG [200 μM]
**11**	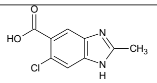	33 %
**12**	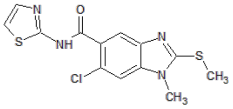	30%
**13**	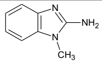	27%
**14**	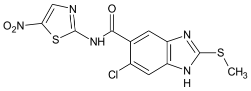	26 %
**15**	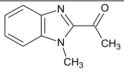	25 %
**16**	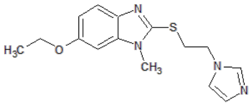	25%
**17**	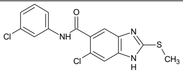	20%
**18**	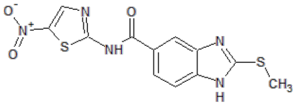	20%
**19**	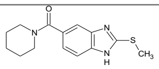	19 %
**20**	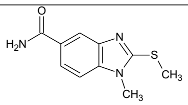	13 %
**21**	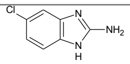	11%
**22**	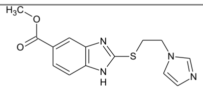	11 %
**23**	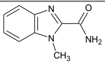	10 %
**24**	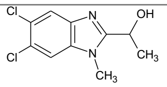	10 %
**25**	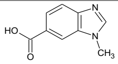	10 %

**26**	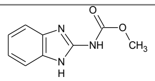	10 %
**27**	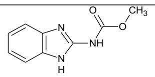	10 %
**28**	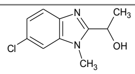	10 %
**29**	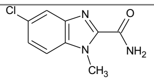	10 %
**30**	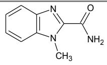	10 %
**31**	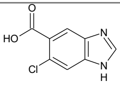	10 %
**32**	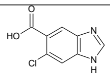	10 %
**33**	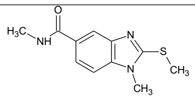	8 %

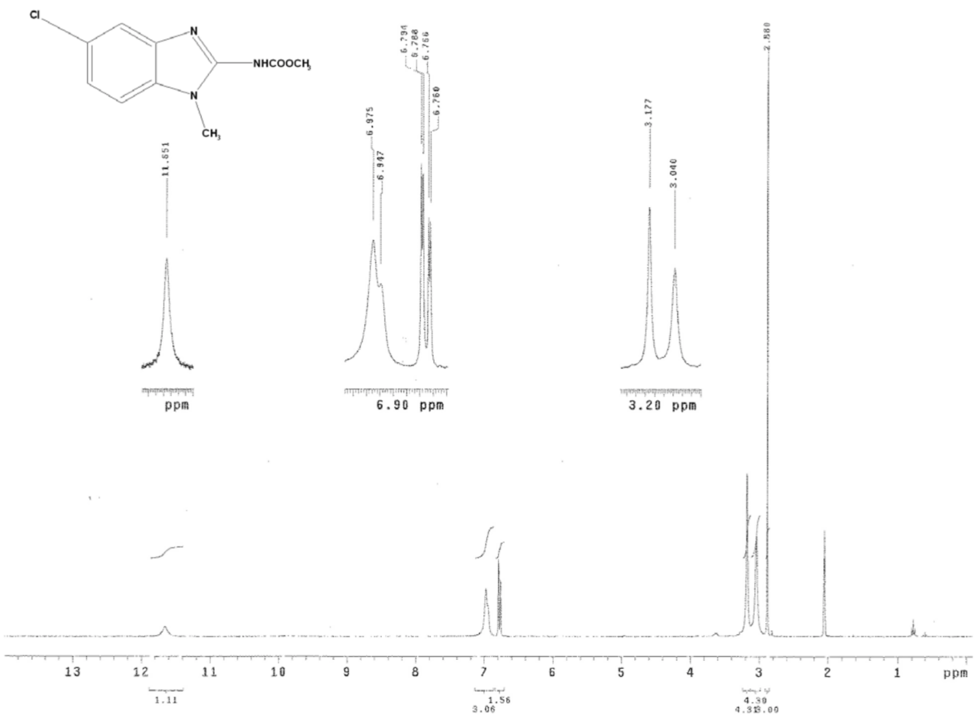

Figure S1. ^{1}H NMR (Acetone-d_6; 300 MHz). Methyl (5-chloro-1-methyl-1*H*-benzimidazol-2-yl)carbamate (**1**).

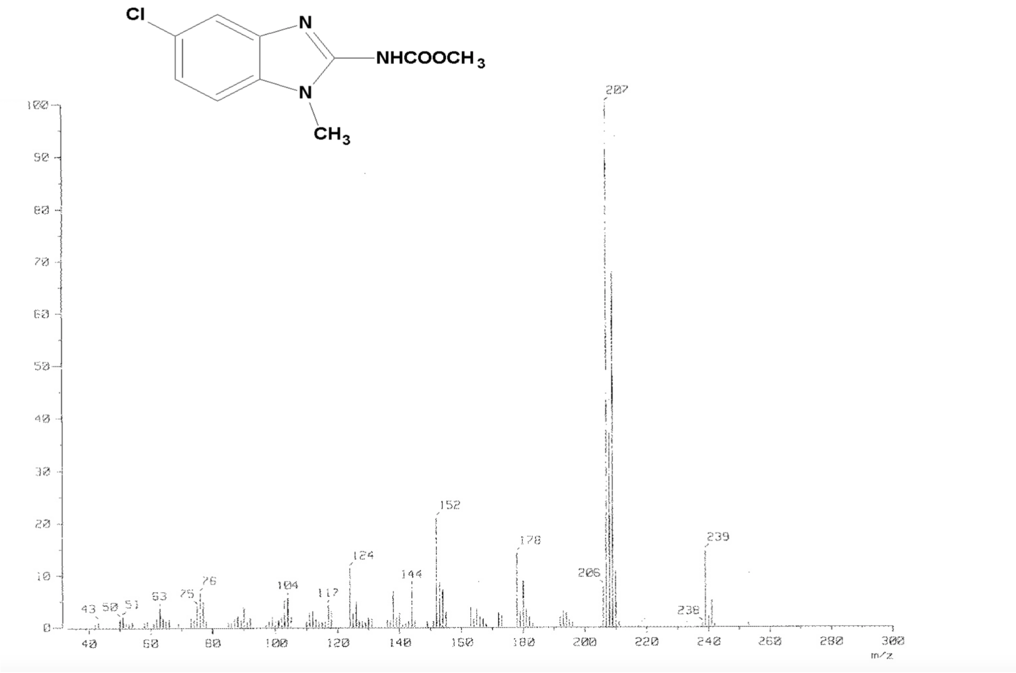

Figure S2. MS (EI$^+$) *m*/*z*: 239 [M]$^+$. Methyl (5-chloro-1-methyl-1*H*-benzimidazol-2-yl)carbamate (**1**).

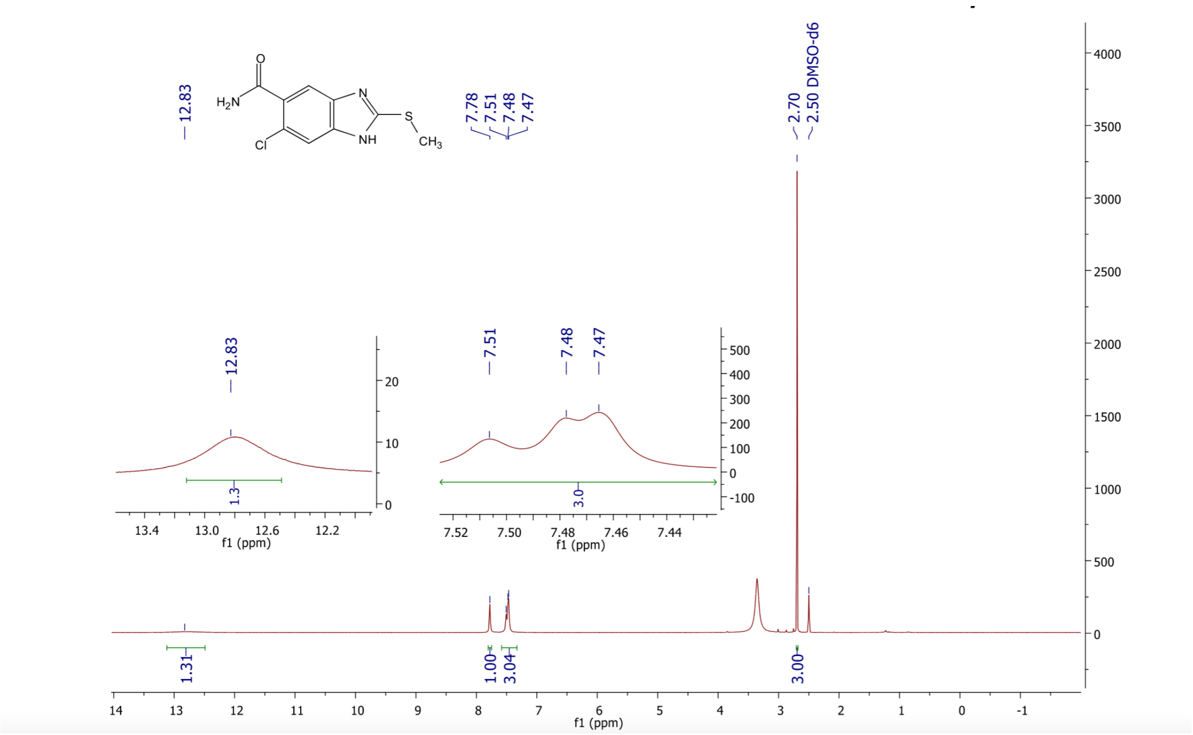

Figure S3. ^{1}H NMR (DMSO-*d*6; 400 MHz). 6-Chloro-2-(methylthio)-1*H*-benzimidazole-5-carboxamide (**2**).

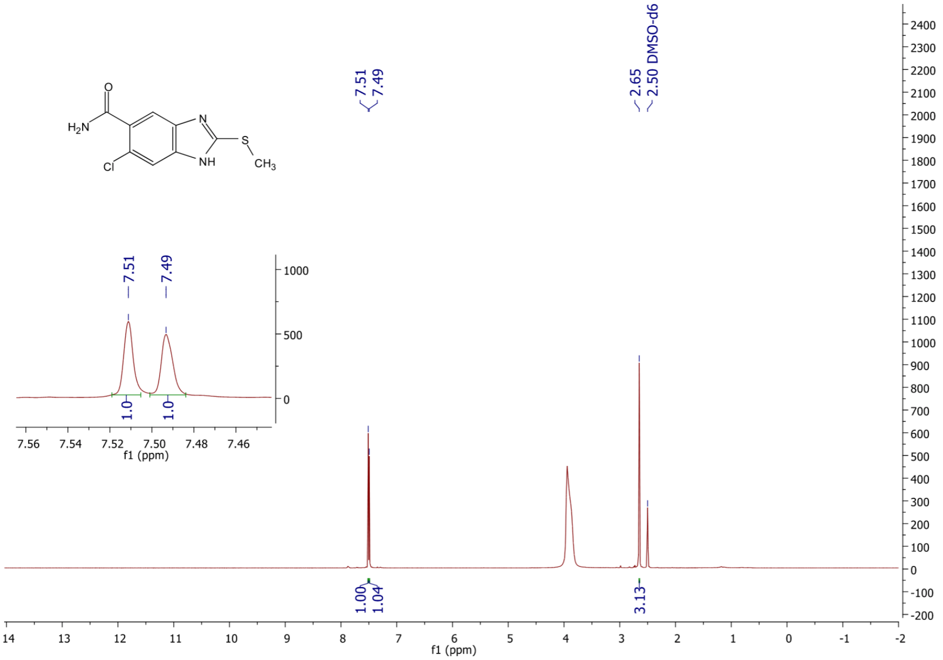

Figure S4. ^{1}H NMR (DMSO-*d*6 + D_2O; 400 MHz). 6-Chloro-2-(methylthio)-1*H*-benzimidazole-5-carboxamide (**2**).

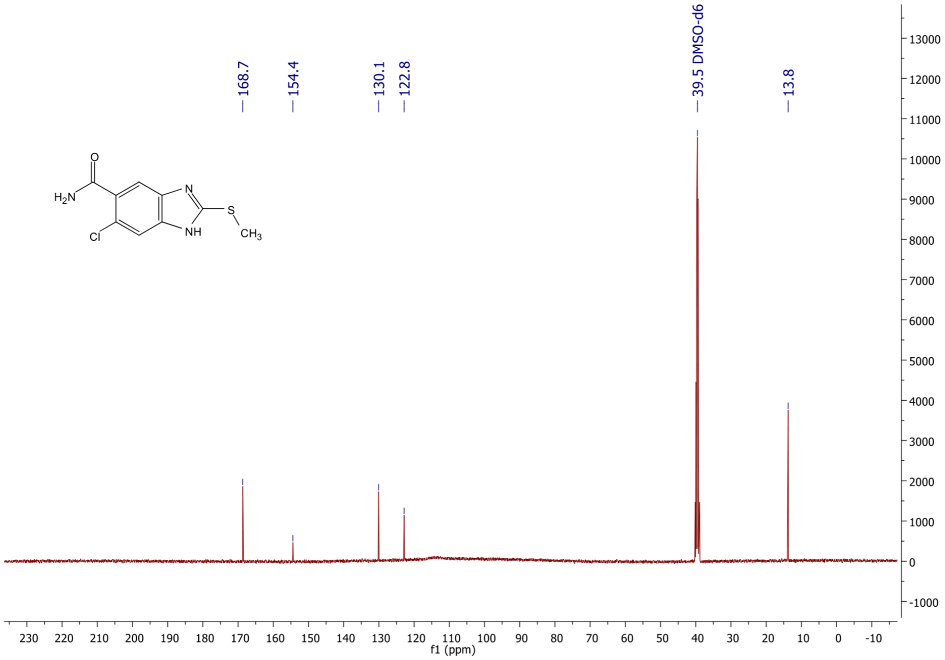

Figure S5. ^{13}C NMR (DMSO-d_6; 100 MHz)6-Chloro-2-(methylthio)-1*H*-benzimidazole-5-carboxamide (**2**).

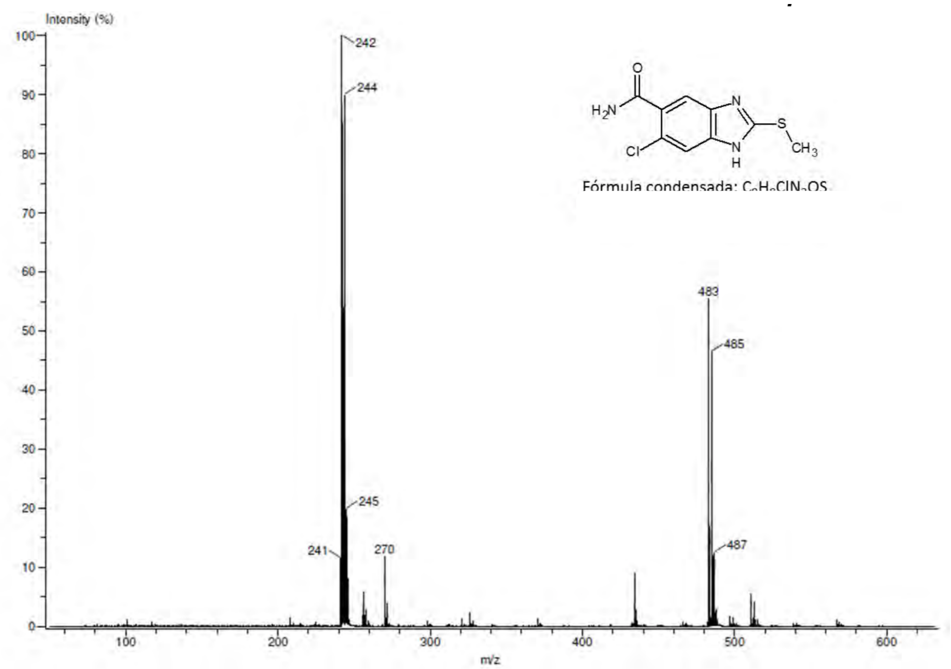

Figure S6. MS (DART$^+$) *m*/*z*: 242 [M+H]$^+$. 6-Chloro-2-(methylthio)-1*H*-benzimidazole-5-carboxamide (**2**).

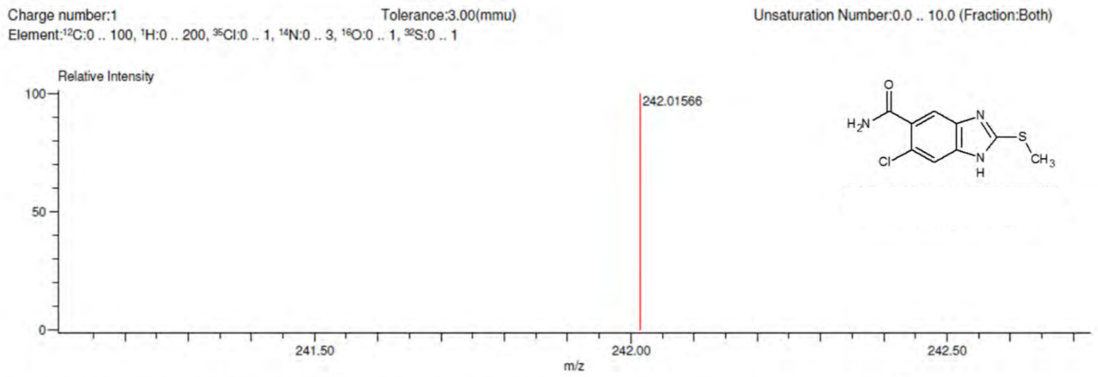

Mass	Intensity	Calc. Mass	Mass Difference (mmu)	Mass Difference (ppm)	Possible Formula	Unsaturation Number
242.01566	1627300.25	242.01549	0.17	0.70	$^{12}C_9{}^1H_9{}^{35}Cl_1{}^{14}N_3{}^{16}O_1{}^{32}S_1$	7.5

Figure S7. HRMS (DART+) cal. [$C_9H_8ClN_3OS$ +H] 242.01549, found 242.01566. 6-Chloro-2-(methylthio)-1*H*-benzimidazole-5-carboxamide (**2**).

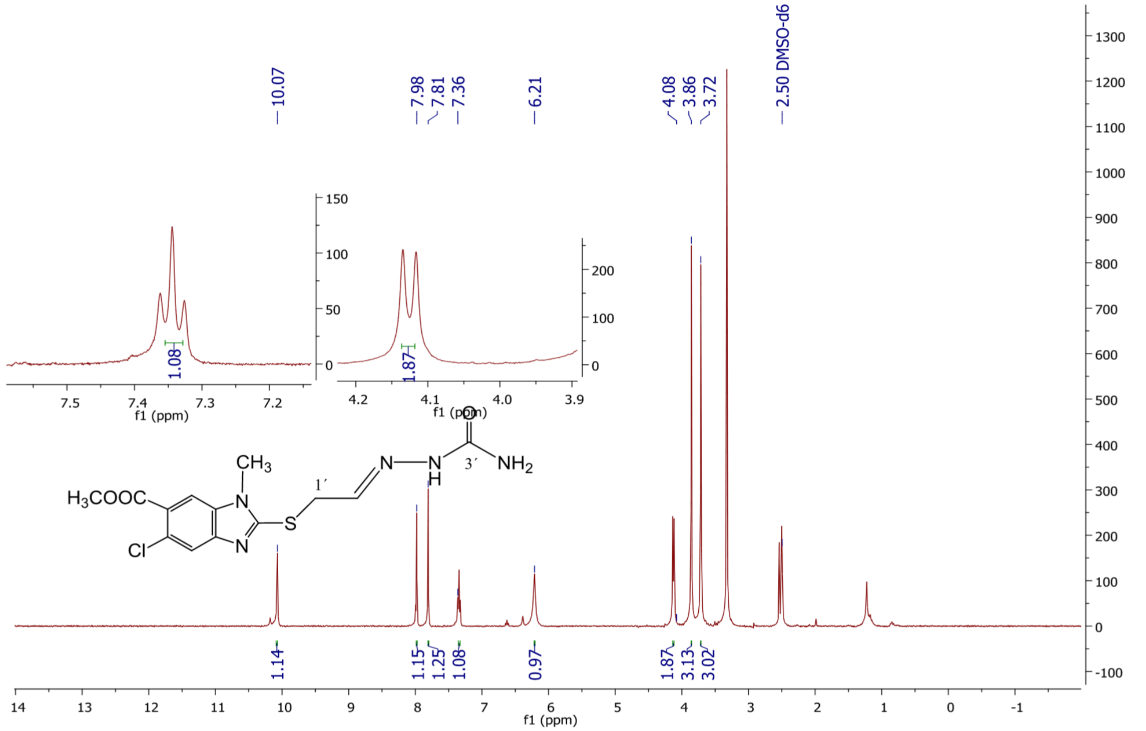

Figure S8. ^{1}H NMR (DMSO-d_6; 300 MHz. Methyl 2-((2-(2-carbamoylhydrazineylidene)ethyl)thio)-5-chloro-1-methyl-1*H*-benzimidazole-6-carboxylate (**3**).

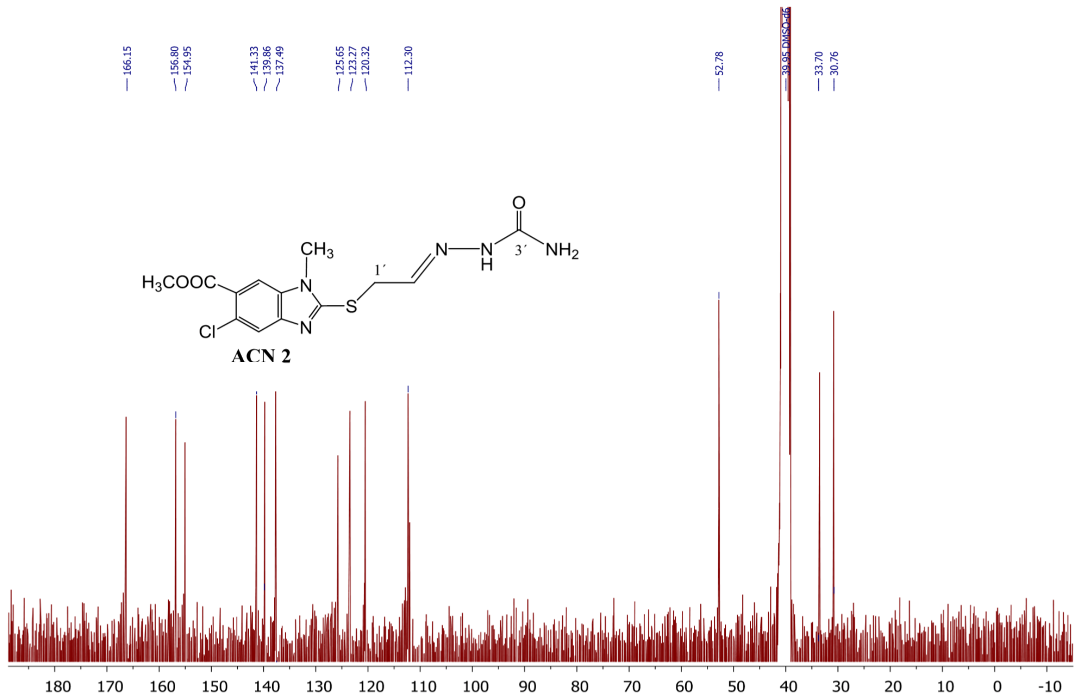

Figure S9. ^{13}C NMR (DMSO-*d*$_6$; 75 MHz). Methyl 2-((2-(2-carbamoylhydrazineylidene)ethyl)thio)-5-chloro-1-methyl-1*H*-benzimidazole-6-carboxylate (**3**).

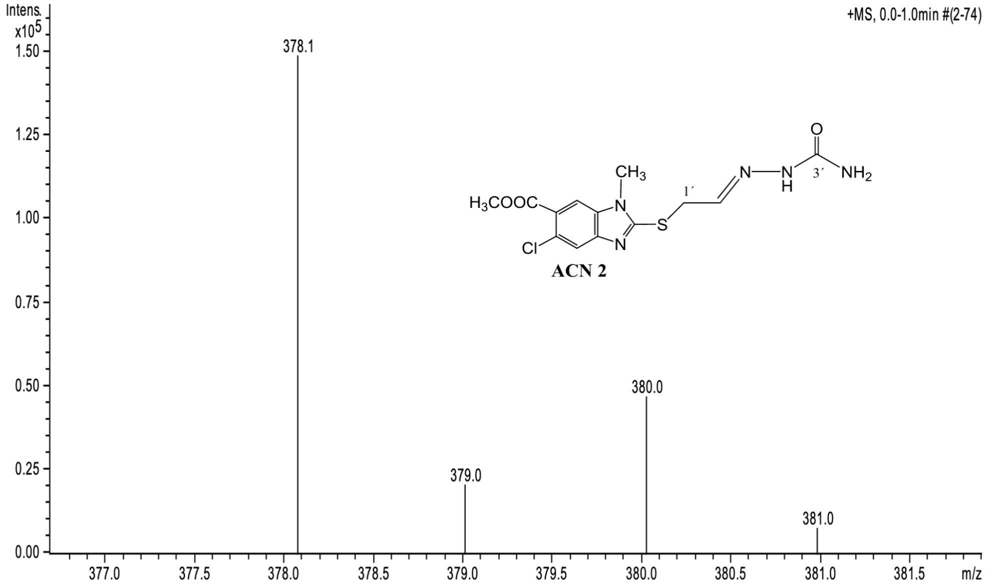

Figure S10. MS (ESI$^+$): *m/z* 378 [M+Na]$^+$. Methyl 2-((2-(2-carbamoylhydrazineylidene)ethyl)thio)-5-chloro-1-methyl-1*H*-benzimidazole-6-carboxylate (**3**).

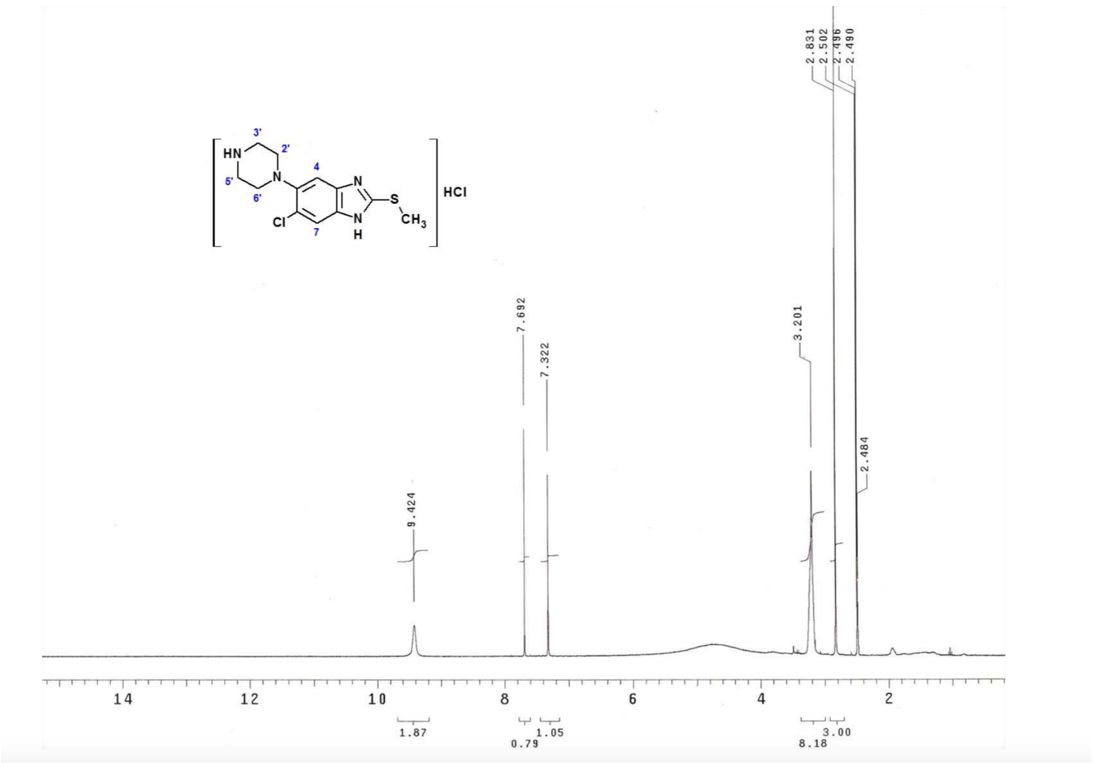

Figure S11. ^{1}H NMR (DMSO-d_6; 300 MHz). 6-Chloro-2-(methylthio)-5-(piperazin-1-yl)-1*H*-benzimidazole (**6**).

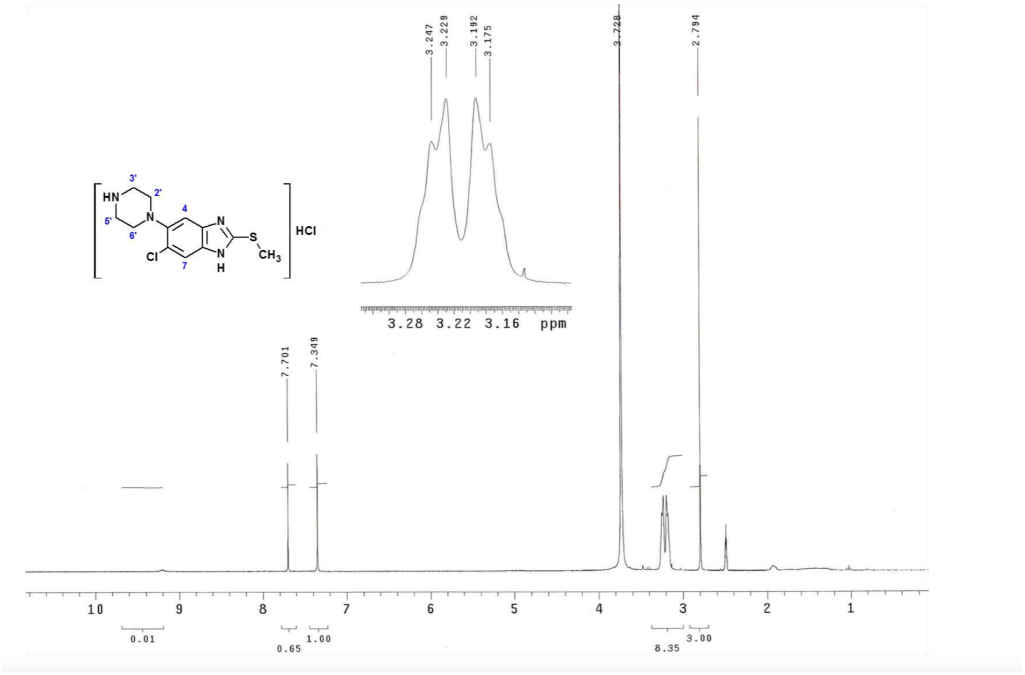

Figure S12. ^{1}H NMR (DMSO-d_6 + D_2O; 300 MHz)6-Chloro-2-(methylthio)-5-(piperazin-1-yl)-1*H*-benzimidazole (**6**).

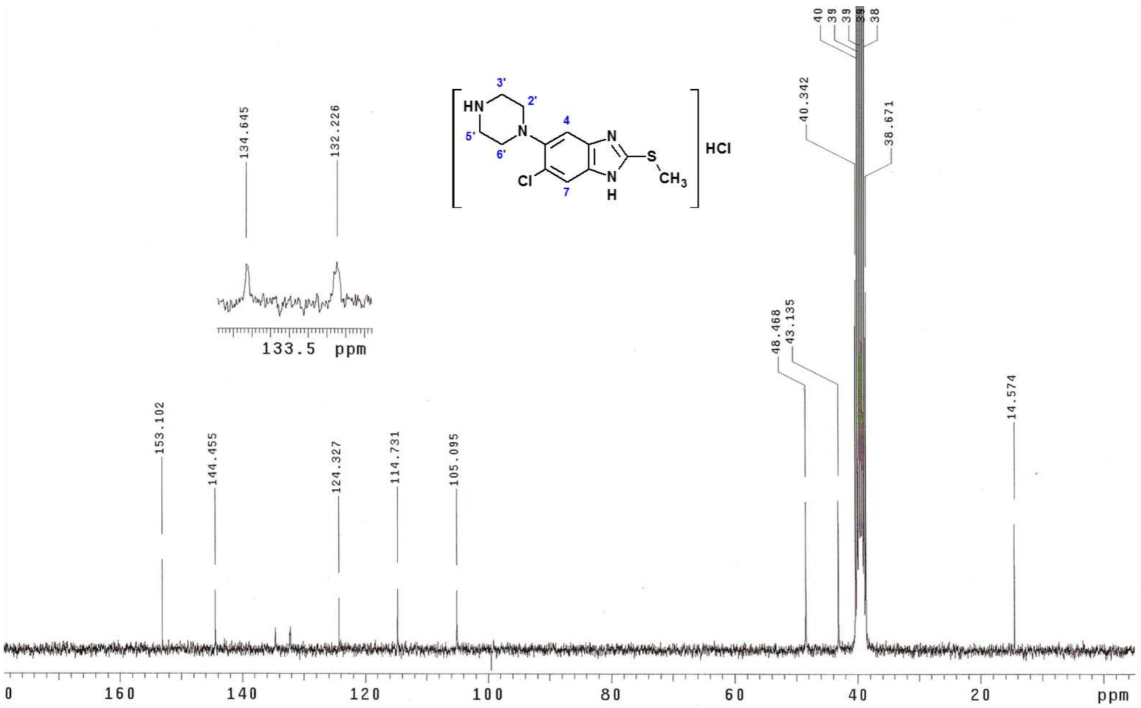

Figure S13.^{13}C NMR (DMSO-d_6; 75 MHz). 6-Chloro-2-(methylthio)-5-(piperazin-1-yl)-1*H*-benzimidazole (**6**).

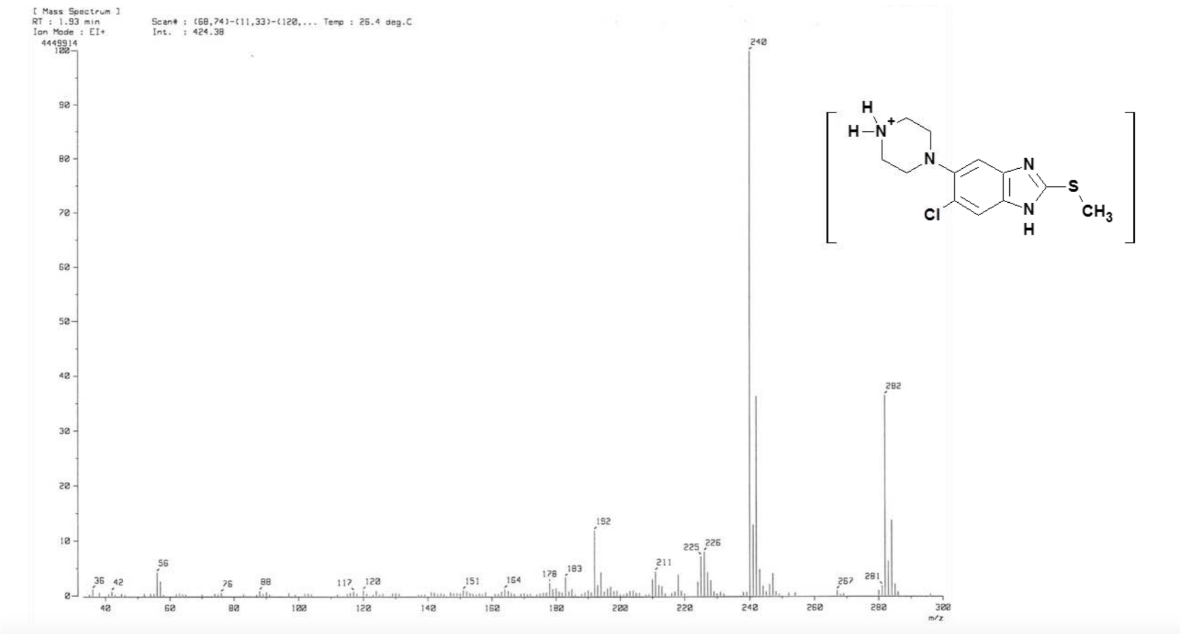

Figure S14. MS (EI$^+$) *m*/*z*: 282 [M]$^+$. 6-Chloro-2-(methylthio)-5-(piperazin-1-yl)-1*H*-benzimidazole (**6**).

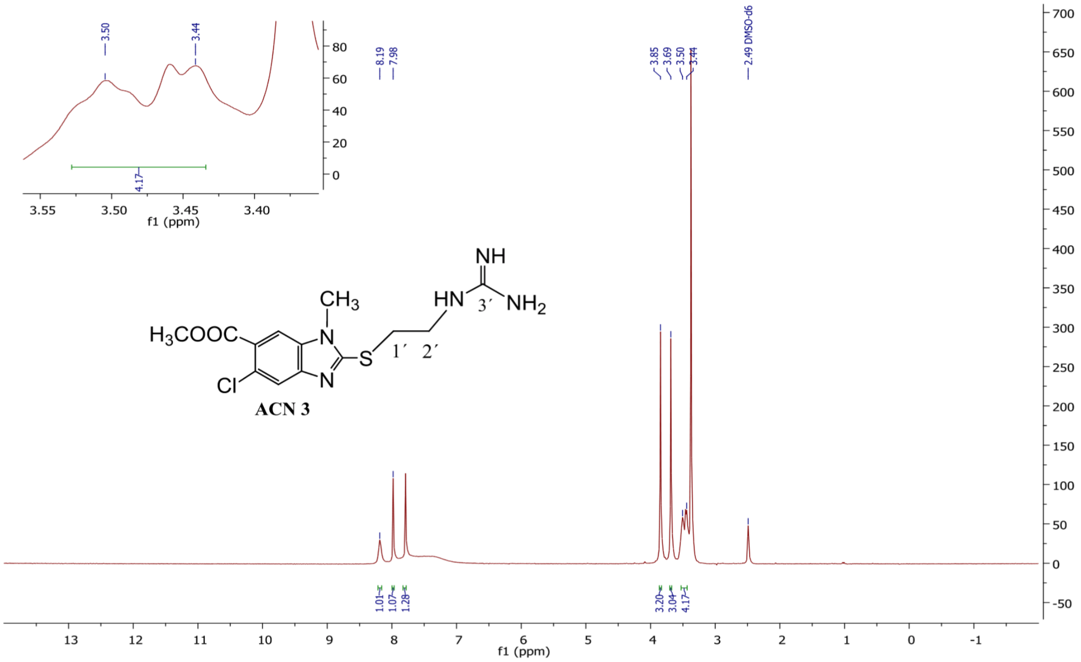

Figure S15. ^{1}H NMR (DMSO-d_6; 400 MHz). Methyl 5-chloro-2-((2-guanidinoethyl)thio)-1-methyl-1*H*-benzimidazole-6-carboxylate (**7**).

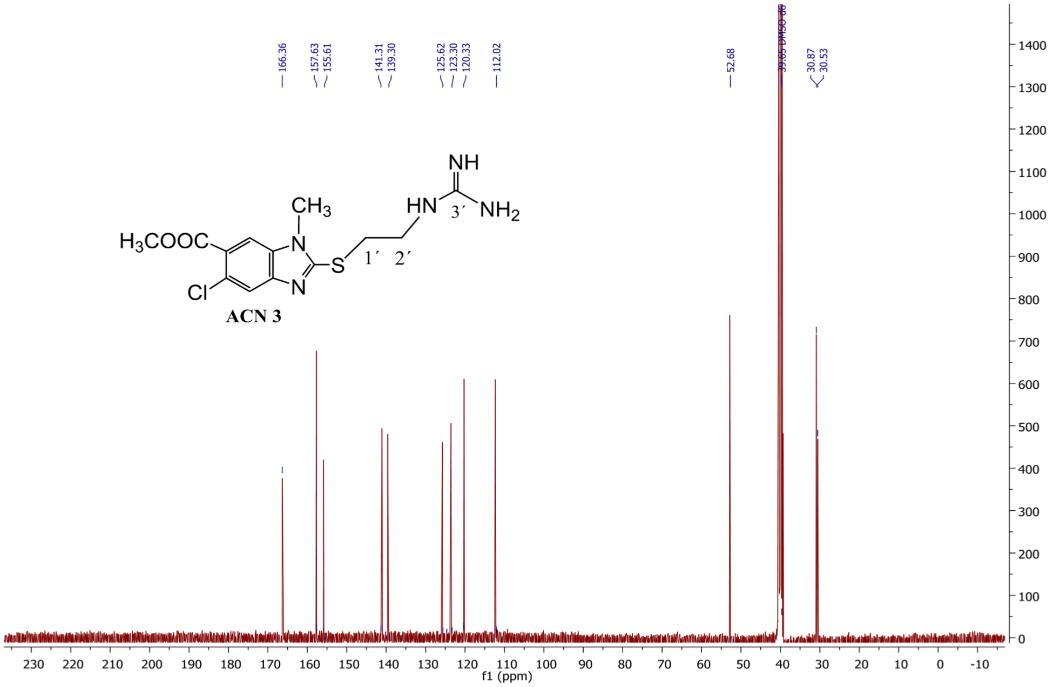

Figure S16. ^{13}C NMR (DMSO-d_6; 100 MHz). Methyl 5-chloro-2-((2-guanidinoethyl)thio)-1-methyl-1*H*-benzimidazole-6-carboxylate (**7**).

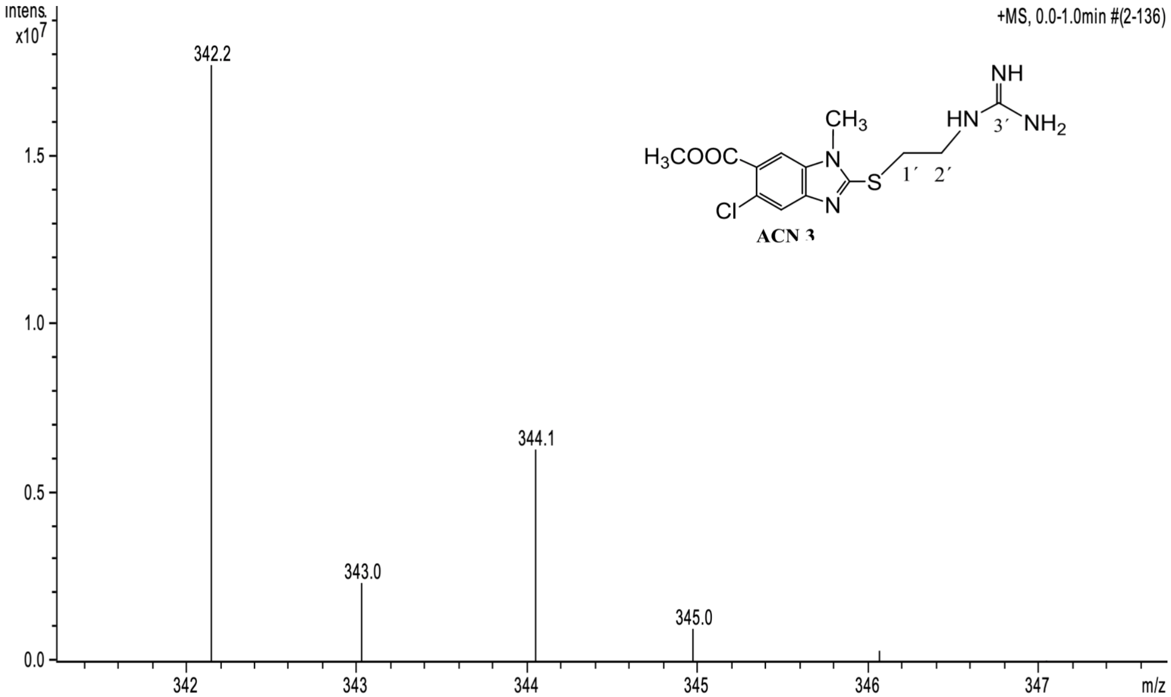

Figure S17. MS (ESI+): *m/z* 342 [M+H]+.Methyl 5-chloro-2-((2-guanidinoethyl)thio)-1-methyl-1*H*-benzimidazole-6-carboxylate (**7**).

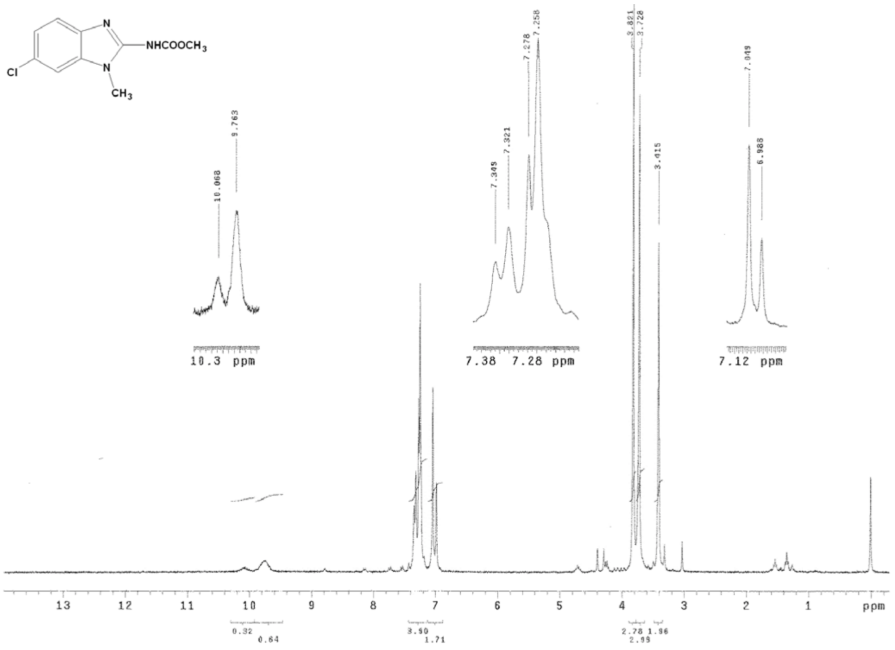

Figure S18.^{1}H NMR ($CDCl_3$; 300 MHz. Methyl (6-chloro-1-methyl-1*H*-benzimidazol-2-yl)carbamate (**8**).

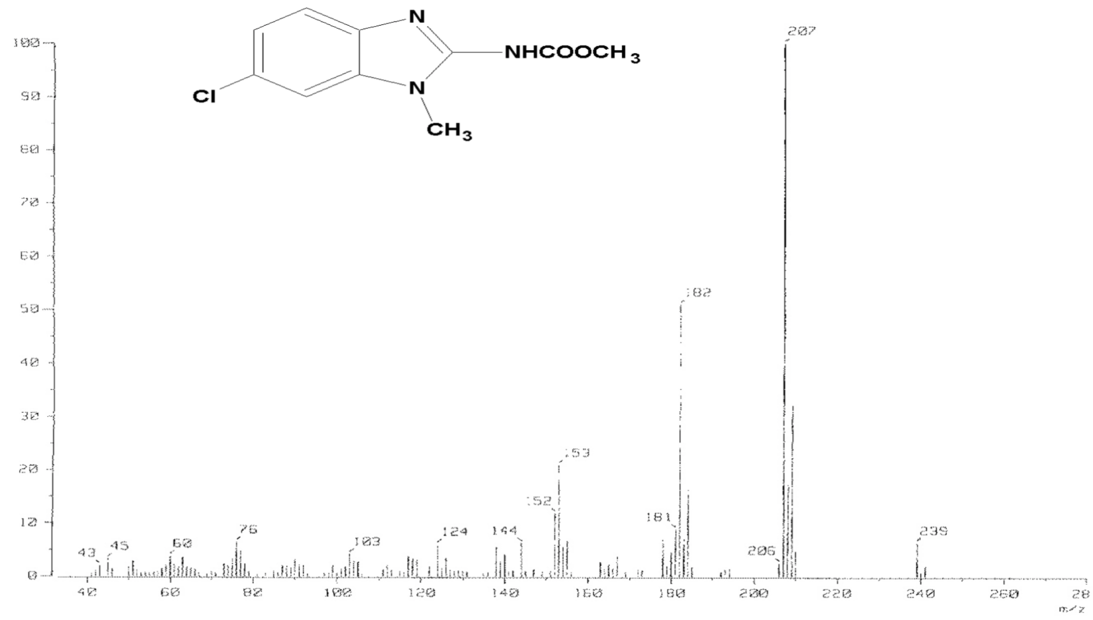

Figure S19. MS (EI+): *m/z* 239 [M]+. Methyl (6-chloro-1-methyl-1*H*-benzimidazol-2-yl)carbamate (**8**).

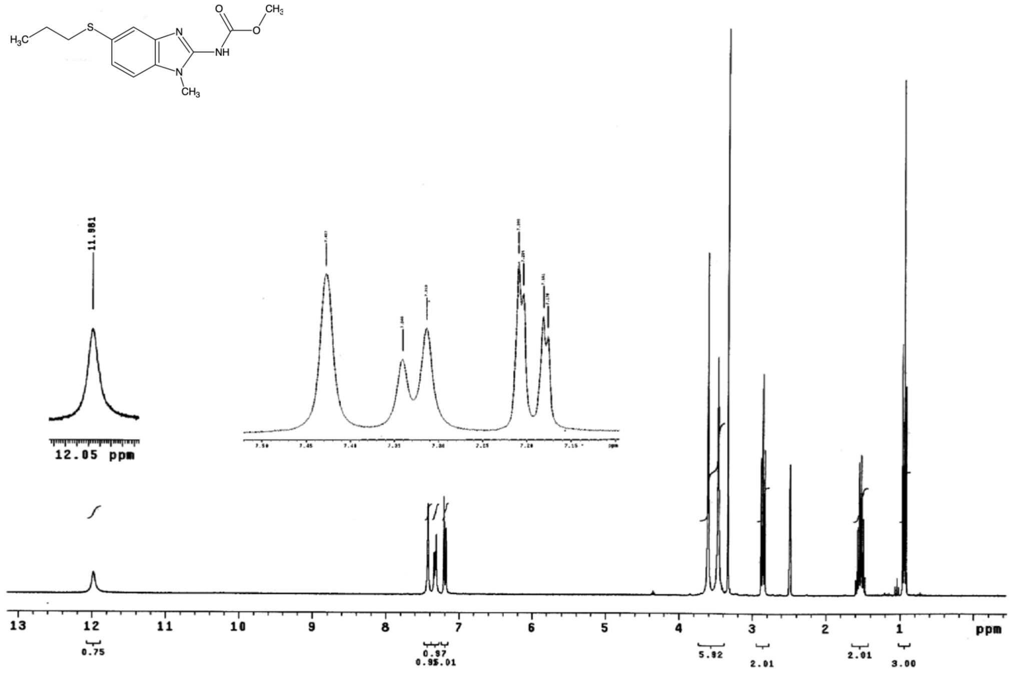

Figure S20. ^{1}H NMR (DMSO-*d*6; 300 MHz). Methyl (1-methyl-5-(propylthio)-1*H*-benzimidazol-2-yl)carbamate (**9**).

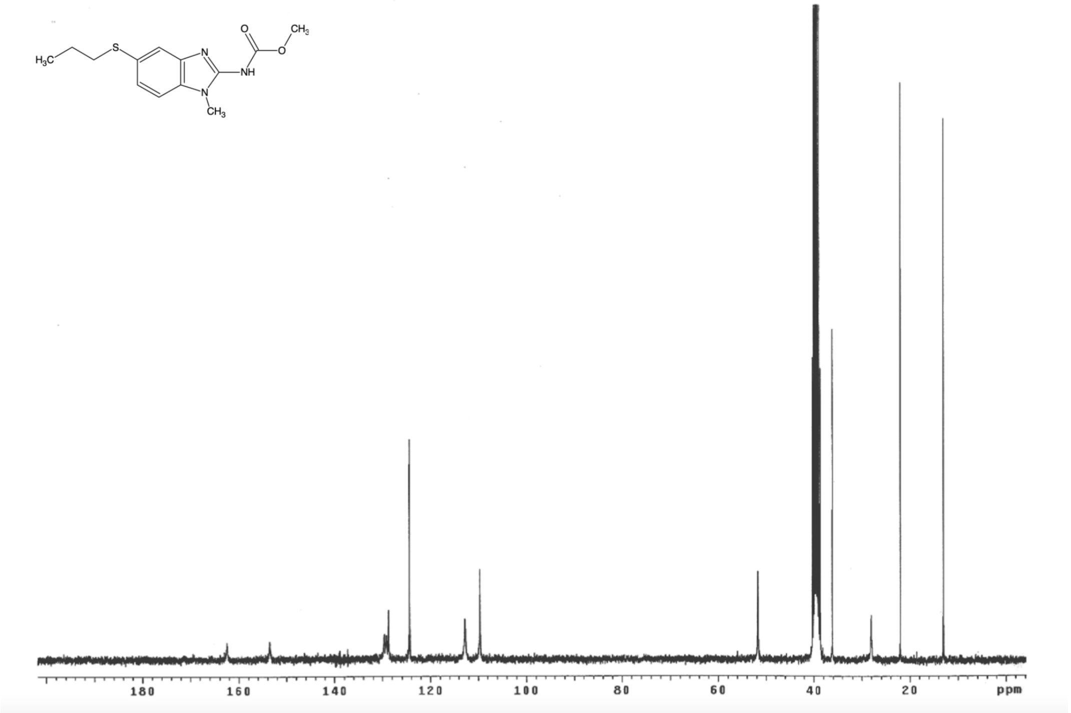

Figure S21.^{13}C NMR (DMSO-d_6; 75 MHz). Methyl (1-methyl-5-(propylthio)-1*H*-benzimidazol-2-yl)carbamate (**9**).

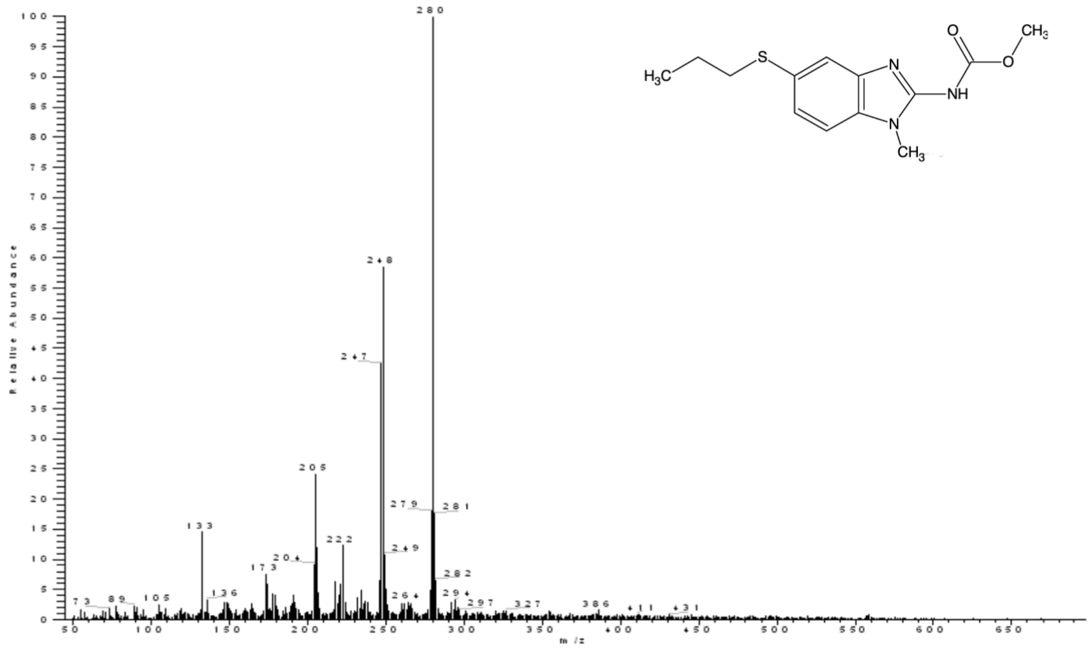

Figure S22. MS (FAB+): *m*/*z* 280 [M+H] +. Methyl (1-methyl-5-(propylthio)-1*H*-benzimidazol-2-yl)carbamate (**9**).
